# Entropy directs the self-assembly of supramolecular palladium coordination macrocycles and cages†

**DOI:** 10.1039/d2sc03154j

**Published:** 2022-08-10

**Authors:** D. A. Poole III, E. O. Bobylev, S. Mathew, J. N. H. Reek

**Affiliations:** Homogeneous, Supramolecular, and Bioinspired Catalysis Group, van ‘t Hoff Institute for Molecular Science (HIMS), University of Amsterdam (UvA) Science Park 904 1098 XH Amsterdam The Netherlands j.n.h.reek@uva.nl

## Abstract

The self-assembly of palladium-based cages is frequently rationalized *via* the cumulative enthalpy (Δ*H*) of bonds between coordination nodes (M, *i.e.*, Pd) and ligand (L) components. This focus on enthalpic rationale limits the complete understanding of the Gibbs free energy (Δ*G*) for self-assembly, as entropic (Δ*S*) contributions are overlooked. Here, we present a study of the M_2_^lin^L_3_ intermediate species (M = dinitrato(*N*,*N*,*N*′,*N*′-tetramethylethylenediamine)palladium(ii), ^lin^L = 4,4′-bipyridine), formed during the synthesis of triangle-shaped (M_3_^lin^L_3_) and square-shaped (M_4_^lin^L_4_) coordination macrocycles. Thermochemical analyses by variable temperature (VT) ^1^H-NMR revealed that the M_2_^lin^L_3_ intermediate exhibited an unfavorable (relative) Δ*S* compared to M_3_^lin^L_3_ (triangle, Δ*T*Δ*S* = +5.22 kcal mol^−1^) or M_4_^lin^L_4_ (square, Δ*T*Δ*S* = +2.37 kcal mol^−1^) macrocycles. Further analysis of these constructs with molecular dynamics (MD) identified that the self-assembly process is driven by Δ*G* losses facilitated by increases in solvation entropy (Δ*S*_solv_, *i.e.*, depletion of solvent accessible surface area) that drives the self-assembly from “open” intermediates toward “closed” macrocyclic products. Expansion of our computational approach to the analysis of self-assembly in Pd_*n*_^ben^L_2*n*_ cages (^ben^L = 4,4'-(5-ethoxy-1,3-phenylene)dipyridine), demonstrated that Δ*S*_solv_ contributions drive the self-assembly of both thermodynamic cage products (*i.e.*, Pd_12_^ben^L_24_) and kinetically-trapped intermediates (*i.e.*, Pd_8_^c^L_16_).

## Abstract 2

These studies demonstrate that Δ*S* drives the self-assembly of supramolecular palladium-based coordination macrocycles and cages. As this Δ*S* contribution arises from solvation, these findings broadly reflect the thermodynamic drive of self-assembly to form compact structures.

## Graphical abstract

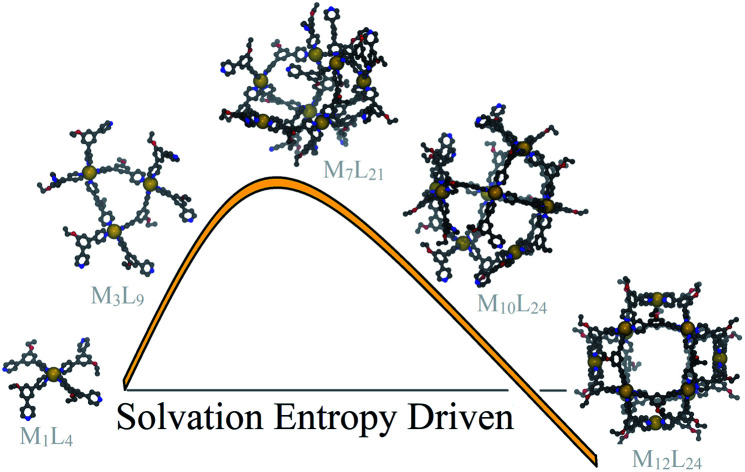

## Introduction

Self-assembled macrocycles,^1–7^ cages,^8–13^ and oligomers^14–16^ featuring palladium-coordination nodes are a mainstay of supramolecular chemistry due to their unique mechanical,^14–18^ optoelectronic,^19–28^ and catalytic^29–42^ applications. The square-planar coordination geometry of the coordination node permits the synthesis of well-defined constructs invoking simple geometric design principles.^43–48^ These constructs are formed by the spontaneous self-assembly of palladium-based coordination nodes with ditopic ligands, affording highly-ordered assemblies bearing the minimum Gibbs free energy (Δ*G*).^43–50^ The synthesis of these assemblies from their many constituent components is rationalized by the cumulative enthalpic (Δ*H*) contributions as a result of the formation of dative bonds between ditopic ligands and Pd-based coordination nodes.^49,50^ However, studies of gas-phase reactivity reveal these Δ*H* contributions decrease with sequential coordination of additional ligands, favoring the formation of coordinatively unsaturated oligomers.^51^ In addition, currently only entropic contributions (Δ*S*) based on statistical arguments are taken into account, implying that assemblies based on a large number of components are entropically largely unfavorable despite many synthetic examples. Whereas similar enthalpic arguments rationalize the difference in energy between various assemblies, it remains unclear why well-defined structures are favored over oligomers as these coordination bonds in principle can be formed with limited constraints. The current rationale for deducing self-assembly thermodynamics is therefore incomplete, which motivates our current study.

**Scheme 1:**
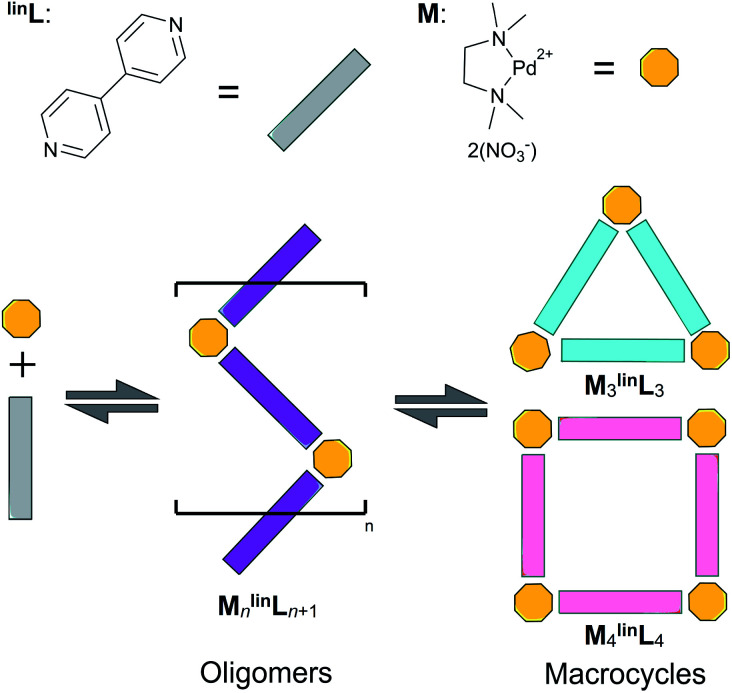
Self-assembly of dinitrato(*N*,*N*,*N*′,*N*′-tetramethylethylenediamine) palladium(ii) (M, yellow) and 4′4-bipyridine (^lin^L, grey) affording a mixture of triangular (M_3_^lin^L_3_, blue) and square (M_4_^lin^L_4_, pink) macrocycles alongside oligomer intermediates (M_*n*_^lin^L_*n*+1_, purple).

The self-assembly of equimolar amounts of dinitrato(*N*,*N*,*N*′,*N*′-tetramethylethylenediamine)palladium(ii) (M) and 4,4′-bipyridine (^lin^L) affords a mixture of triangular and square macrocycles in equilibrium (Scheme 1).^1,2^

Previous reports have leveraged this experimentally accessible equilibrium to measure the relative Δ*S* and Δ*H* of triangular and square complexes.^5^ These studies found that Δ*S* favors the assembly featuring fewer components (*i.e.*, triangles) while Δ*H* favors the geometric matching between the square-planar metal center and the ligand geometry (*i.e.*, squares).^1,5^ The synthesis of (and conversion between) macrocyclic assemblies proceeds *via* coordinatively unsaturated oligomeric intermediates (Scheme 1, purple).

Interestingly, similar stable oligomer intermediates have been found in the synthesis of polygonal organometallic macrocyclic assemblies.^1–5^ The equilibrium between these stable oligomeric intermediates and macrocyclic products may be leveraged to quantify Δ*H* and Δ*S* contributions to self-assembly using the literature described NMR-based approach.^5^ Realization of the origin and effect of these thermodynamic contributions enables rational improvement of the self-assembly of highly-ordered constructs used broadly in supramolecular chemistry, including coordination cages, metal–organic frameworks, and dynamic-covalent based constructs.^52,59–62^

**Scheme 2:**
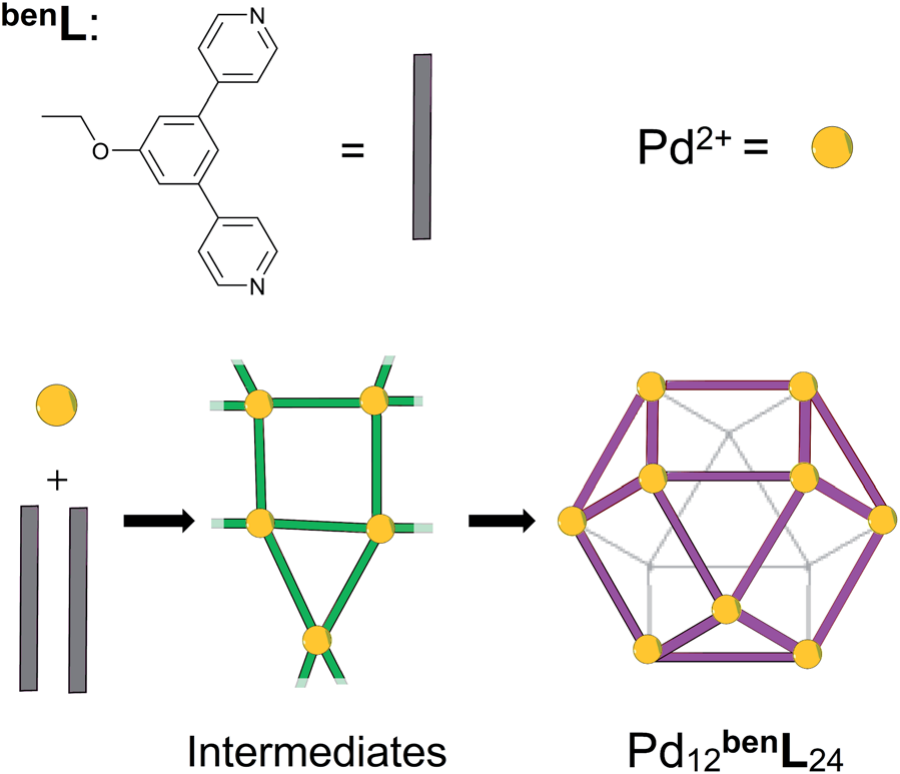
The self-assembly of Pd_12_^ben^L_24_ cages (^ben^L = 4,4′-(5-ethoxy-1,3-phenylene)dipyridine) *via* poorly defined reticular intermediates (green).

In this report, we demonstrate that the self-assembly of an equimolar mixture of M and ^lin^L in deuterated dimethyl sulfoxide (DMSO) affords a mixture of triangular (M_3_^lin^L_3_) and square (M_4_^lin^L_4_), and oligomeric (M_*n*_^lin^L_*n*+1_) assemblies as depicted in Scheme 1.^1,6^ We employed variable temperature ^1^H-NMR (VT-NMR) to determine the Δ*H* and Δ*S* of both oligomeric and macrocyclic assemblies, providing unprecedented thermodynamic insights into the self-assembly process. Importantly, this thermodynamic data-enabled validation of a molecular dynamics (MD)^52^ based approach to distinguish respective Δ*S* contributions arising from the assembly structure (Δ*S*_struct_, eqn S1†)^53^ and its solvation (Δ*S*_solv_, eqn S2†).^54^ These individual entropic contributions, alongside calculation of Δ*H* (eqn S3†), ultimately provide an accurate Δ*G* for self-assembly. We applied our MD-based approach to the study of coordination cages based on 4,4'-(5-ethoxy-1,3-phenylene)dipyridine as a bent ditopic ligand (^ben^L) and free palladium(ii) ions (Pd^2+^), which have been reported in the literature (Scheme 2).^8–13^ Thermodynamic estimates derived from MD simulations reveal a Δ*S*_solv_-driven, self-assembly process for macrocycles and cages reminiscent of biopolymer folding.^51^ The generalization of our MD-based approach may distinguish between kinetically accessible thermodynamic products (*i.e.*, Pd_12_^ben^L_24_) and undesirable kinetically-trapped intermediate assemblies (*e.g.* Pd_8_^ben^L_16_).^55^

These computational and experimental studies demonstrate that Δ*S* drives the self-assembly of supramolecular constructs featuring palladium coordination nodes. As this Δ*S* contribution arises from solvation, these findings broadly reflect the thermodynamic drive of self-assembly to form compact supramolecular structures. Furthermore, we demonstrate the utility of MD-based approaches to quantify the thermodynamics of large supramolecular systems, providing a methodology that enables *in silico* studies of self-assembly processes.

## Results

### Synthesis and characterization of assemblies based on M and linL

**Fig. 1:**
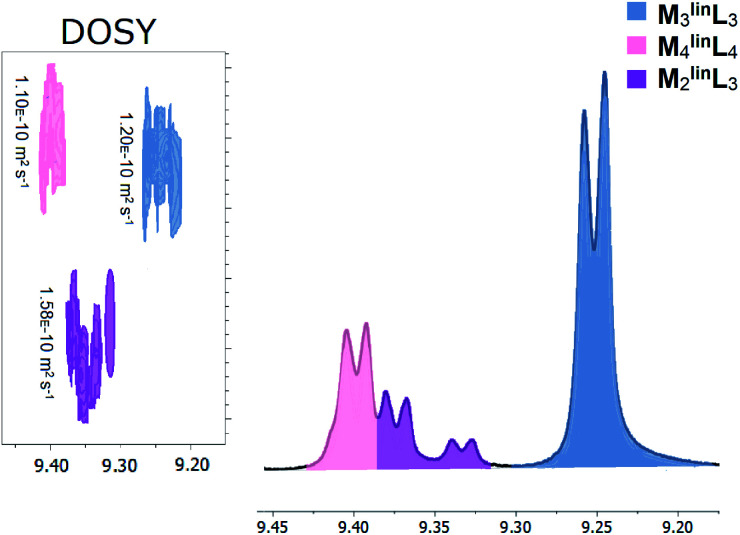
Macrocyclic and oligomeric products observed from the self-assembly of M (17.1 mM) and ^lin^L (17.1 mM) in DMSO characterized by unique α-pyridyl peaks in ^1^H-NMR (Fig. S1†), with inset DOSY (Fig. S2†) diffusograms. Three distinct assemblies were observed and highlighted for clarity: M_3_^lin^L_3_ (slate blue), M_4_^lin^L_4_ (pink), and M_2_^lin^L_3_ (purple). Numerical data is provided below in Table 1.

Previously, we reported that the absence of trace halide impurities during the self-assembly of coordination cages resulted in slower formation kinetics, giving rise to the observation of intermediates.^49^ Thus, we developed an alternative preparation for M, using a limiting quantity of palladium dichloride to minimize trace chloride (Scheme S1†). With this prepared metal precursor, the self-assembly of stoichiometric quantities of M and ^lin^L (M : ^lin^L = 1.0 : 1.0, [M] = 17.1 mM) affords a mixture of macrocyclic (M_3_^lin^L_3_ and M_4_^lin^L_4_) and oligomeric products evidenced by characteristic ^1^H–NMR peaks shown in Fig. 1.

Peaks corresponding to M_3_^lin^L_3_ (*δ* = 9.24 ppm) and M_4_^lin^L_4_ (*δ* = 9.40 ppm) were consistent with the reported values of these macrocyclic species.^3^ Two additional peaks (*δ* = 9.32–9.37 ppm) present, with chemical shifts consistent with reported oligomers,^1,2^ and a single diffusion constant (*D* = 1.56 × 10^−10^ m^2^ s^−1^, Fig. 1 inset). These features indicate the presence of a single coordination assembly with a size larger than free ^lin^L (*D* = 1.86 × 10^−10^ m^2^ s^−1^) but smaller than M_3_^lin^L_3_ (*D* = 1.20 × 10^−10^ m^2^ s^−1^). We also observed a near 2 : 1 ratio of α-pyridyl peak areas (Table 1), assuming the overlap of α-pyridyl protons adjacent to coordination, we assigned these peaks to an oligomer species with the composition M_2_^lin^L_3_.

**Table 1:** ^1^H-NMR characterization of assemblies based on M and ^lin^L^a^

Assembly	*δ*_α-pyridyl_ (ppm)	*δ*_β-pyridyl_ (ppm)	% Area (α-pyridyl)	Diffusion, *D* (× 10^−10^ m^2^ s^−1^)
M_3_^lin^L_3_	9.24	8.28	63.9	1.20
M_4_^lin^L_4_	9.40	8.21	21.2	1.10
M_2_^lin^L_3_^b^	9.37	8.24	9.9	1.58
M_2_^lin^L_3_^c^	9.32	8.19	5.0	1.58
^lin^L^d^	8.75	7.86	—	1.86

a ^1^H-NMR Conditions: DMSO, 300 K, 300 MHz.

b Outer ligands, *i.e.*, ^lin^L-M-^lin^L-M-^lin^L.

c Internal ligand, *i.e.*, ^lin^L-M-^lin^L-M-^lin^L.

d Reference values were obtained separately for free ^lin^L (Fig. S3).

While previous studies rationalized that the self-assembly process is driven to maximize the number of coordination bonds formed, affording coordinatively saturated species that minimize the Δ*H* of the system.^1^ However, analysis by Weilandt *et al.* on mononuclear Pd complexes demonstrated that the formation of successive coordination bonds result in diminishing Δ*H* contributions to the Δ*G* of complex formation, which was partially compensated by Δ*S*.^56^ Our observation of a significant presence of oligomeric (coordinatively unsaturated) assemblies (14.9%, Table 1), we infer that Δ*S* may play a similarly significant role in macrocycle assembly.

### Thermochemical analysis of macrocycle–oligomer equilibria

**Fig. 2:**
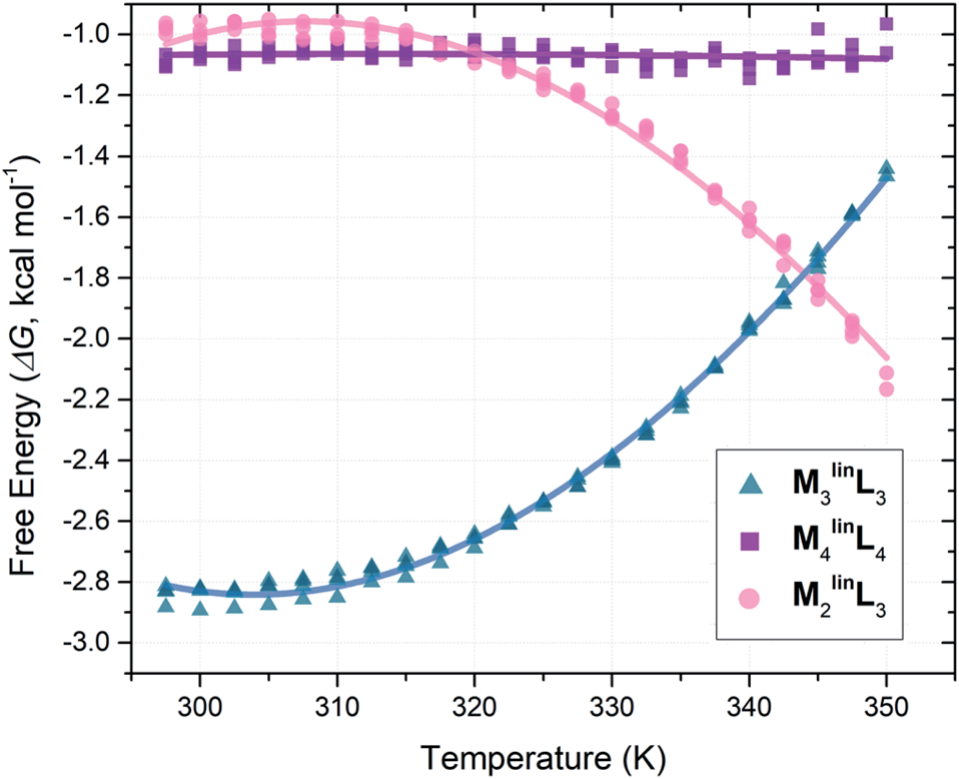
Plot of Δ*G versus* temperature plot from VT-NMR measurements (^1^H = 300 MHz, DMSO, *T*_r_ = 30 s) from quadruplicate temperature sweeps. Free energy values (Δ*G*) were computed from peak areas (Table 1) with a simplified reaction model (Scheme S2†).

Following the literature, we employed VT-NMR to quantify the relative abundance of assemblies (M_2_^lin^L_3_, M_3_^lin^L_3_, and M_4_^lin^L_4_) by monitoring the intensity of their unique α-pyridyl peaks (Table 1) over a wide range of temperatures (297.5–350.0 K, see Fig. S12–S16†).^5^ To determine the relative Δ*G* of M_2_^lin^L_3_, M_3_^lin^L_3_, and M_4_^lin^L_4_ we modeled the system as three orthogonal equilibria between each product and a common pool of reactants (Scheme S2†). These relative Δ*G* values (Table S1†) were plotted as a function of temperature (Fig. 2), and directly fit with an expanded van ‘t Hoff equation to compute Δ*S* and Δ*H* (Scheme S2†).^58^

Consistent with literature observations, these experimentally established Δ*S* and Δ*H* values confirm that M_3_^lin^L_3_ and M_4_^lin^L_4_ are the respective Δ*S*- and Δ*H*-favored macrocyclic products.^5^ Interestingly, M_2_^lin^L_3_ features a lower Δ*H* than the Δ*H*-favored M_4_^lin^L_4_ (Table 2, ΔΔ*H* = −2.27 kcal mol^−1^) and an elevated −*T*Δ*S* compared to the Δ*S*-favored M_3_^lin^L_3_ (Table 2, Δ*T*Δ*S* = +5.22 kcal mol^−1^). These results are contrary to established geometric and component-number based rationale for Δ*H*- and Δ*S*-favored products.^1,4,5^

**Table 2:** Experimental thermodynamic differences between M_2_^lin^L_3,_M_3_^lin^L_3,_ and M_4_^lin^L_4_ coordination assemblies

Assembly	Relative thermodynamic parameters (kcal mol^−1^)^a^		
	Δ*H*°	−*T*Δ*S*°	Δ*G*°
M_3_^lin^L_3_	0.00 ± 0.20	−2.80 ± 0.19	−2.80 ± 0.02
M_4_^lin^L_4_	−1.11 ± 0.29	+0.05 ± 0.28	−1.06 ± 0.03
M_2_^lin^L_3_	−3.38 ± 0.34	+2.42 ± 0.32	−0.96 ± 0.31

a Values determined for *T* = 298.15 K by direct fitting a modified van ‘t Hoff model (Scheme S2) to VT-NMR derived Δ*G* values (Fig. 2).^58^

We surmise that the Δ*H* of M_2_^lin^L_3_ derives from the conformational freedom of the structure, allowing the adoption of an unstrained configuration (∠N–Pd–N = 90°) following the geometric rationale established for M_4_^lin^L_4_. However, M_4_^lin^L_4_ exhibits an internal strain relative to M_2_^lin^L_3_ manifesting as the Δ*H*-difference between the two complexes (ΔΔ*H* = −2.27 kcal mol^−1^). As both assemblies are presumed to adopt a conformation where ∠N–Pd–N = 90°, the apparent ΔΔ*H* is not accounted for when a simple geometric rationale is invoked.^1^ Moreover, Δ*S*-differences between the 6-component M_3_^lin^L_3_ and 5-component M_2L3_ contrast the typical rationale, which correlates the integration of fewer components to a favorable Δ*S*. These findings highlight how the current rationale for determining Δ*S* and Δ*H* contributions is insufficient to account for oligomeric assemblies, necessitating further computational investigation into the origins of internal strain found in M_4_^lin^L_4_ and the unexpected Δ*S* penalties associated with M_2_^lin^L_3_ formation.

### MD analysis of experimental assemblies

**Fig. 3:**
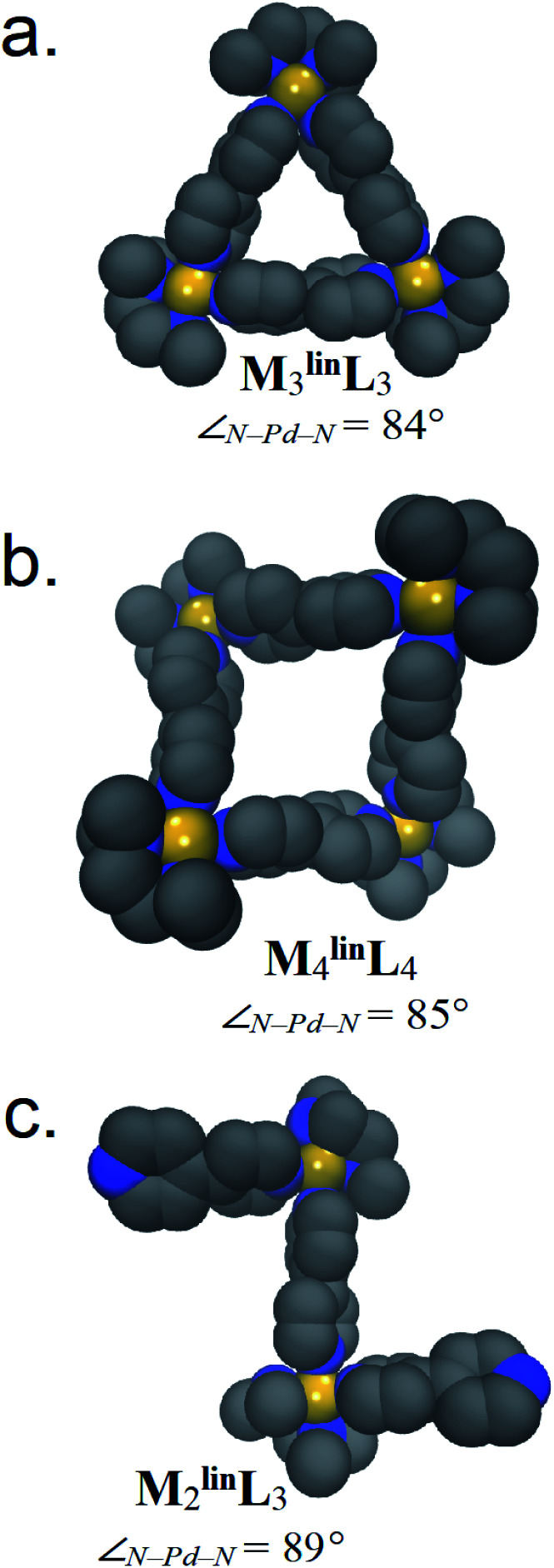
Representative structures of (a) M_3_^lin^L_3_, (b) M_4_^lin^L_4_, and (c) M_2_^lin^L_3_ are rendered with van der Waals representations of the non-hydrogen atoms. Relative thermodynamic contributions to Δ*G* are listed below in Table 3. Adjacent to each model is the average ∠N–Pd–N observed during MD.

Weilandt *et al.* suggested that two discrete Δ*S* factors that exert significant influence on metal–organic complex formation. The first is Δ*S*_struct_, which decreases as more molecules (*i.e.*, components) are required to form a complex.^5^ The other is Δ*S*_solv_, which decreases as more solvent molecules are required to solvate a complex.^56^ Using a previously described methodology,^52^ we developed parameters to simulate assemblies comprised of M and ^lin^L with accurate Δ*H* contributions (Fig. S4†). Using these parameters, and GBSA model solvation,^54^ 50 ns trajectories (Fig. S5–S7†) were propagated by MD for M_2_^lin^L_3_, M_3_^lin^L_3_, and M_4_^lin^L_4_ assemblies (Fig. 3). These trajectories were then used to compute the Δ*S*_struct,_^53^ Δ*S*_solv_,^54^ and Δ*H* contributions to Δ*G* (Table 3).

**Table 3:** Thermodynamic values of assemblies based on M and ^lin^L computed from MD trajectories

Assembly	Relative thermodynamic parameters (kcal mol^−1^)			
	Δ*H*°	−*T*Δ*S*_struct_°	−*T*Δ*S*_solv_°	Δ*G*°
M_3_^lin^L_3_	+0.16	+4.15	−12.24	−7.93
M_4_^lin^L_4_	−0.32	+6.15	−12.16	−6.33
M_2_^lin^L_3_	−2.52	+1.30	−4.95	−6.17

The thermodynamic contributions to Δ*H* and Δ*S* computed from these simulations (Table 3) differ those obtained by VT-NMR (Table 2) in their absolute value. However, the differences (*i.e.*, ΔΔ*G*, ΔΔ*H*, or Δ*T*Δ*S*) between assemblies measured by simulation and experiment are very similar (see below). The differences in absolute value reflect the different reference states in experimental and computational measurements (Fig. S20†). The reproduction of the relative differences in these physical quantities validates our *in silico* methodology for the thermodynamic values of these and similar assemblies.

The Δ*H* difference between M_2_^lin^L_3_ and M_4_^lin^L_4_ measured by VT-NMR (ΔΔ*H*_exp_ = +2.28 kcal mol^−1^, Table 2), is similar to our MD-derived results (ΔΔ*H*_MD_ = +2.20 kcal mol^−1^, Table 2). As Δ*H* generally originates from molecular geometry, we infer that the M_4_^lin^L_4_ adopts a geometrically unfavorable (*i.e.*, strained) configuration compared to M_2_^lin^L_3_. Visualization of MD trajectory data for M_2_^lin^L_3_ assemblies reveals that this oligomer prefers a zig-zag conformation with a near-ideal square-planar coordination geometry at the Pd center (∠N–Pd–N = 89°, Fig. 3c). In contrast, visualization of M_4_^lin^L_4_ reveals a folded-square structure that features a hyperbolic geometry (*i.e.*, ∠N–Pd–N = 86°, Fig. 3b) giving rise to an internal strain that is enthalpically unfavorable (*i.e.*, elevates Δ*H*). Additional simulations of M_4_^lin^L_4_ (performed *in vacuo*) reinforce that these distortions are a consequence of the solvation incurred to minimize the solvent-accessible surface area (Fig. S8†).

The *T*Δ*S* difference between M_4_^lin^L_4_ and M_2_^lin^L_3_ measured by VT-NMR (Δ*T*Δ*S*_exp_ = +2.36 kcal mol^−1^, Table 2), is similar to our MD-derived results (Δ*T*Δ*S*_MD_ = +2.37 kcal mol^−1^, Table 2), while other comparisons values have acceptable deviation (Fig. S20†). The calculated −*T*Δ*S*_struct_ values from our MD-approach decrease with the number of components (*i.e.*, M_2_^lin^L_3_ < M_3_^lin^L_3_ < M_4_^lin^L_4_), in line with reported trends.^1,5^ While, the computed difference of Δ*S*_struct_ for M_3_^lin^L_3_ and M_4_^lin^L_4_ are similar to experimental values (Table 2), differences between both macrocycles *vs.*M_2_^lin^L_3_ deviate significantly (Table 3). The inclusion of −*T*Δ*S*_solv_ improves the accounting for Δ*S* differences between M_2_^lin^L_3_, M_3_^lin^L_3_, and M_4_^lin^L_4_ assemblies (Table 3). This leads us to infer that experimental −*T*Δ*S* penalties associated with M_2_^lin^L_3_ formation originate from these Δ*S*_solv_ contributions, in agreement with thermodynamic studies of mono-nuclear Pd complexes.^56^

These thermodynamic parameters demonstrate that Δ*S*-specifically Δ*S*_solv_-drives the conversion of oligomeric intermediates (*i.e.*, M_2_^lin^L_3_) to their macrocyclic product assemblies (*i.e.*, M_3_^lin^L_3_ and M_4_^lin^L_4_). Moreover, the effect of Δ*S*_solv_ may overcome Δ*H* contributions, resulting in strained and distorted molecular geometries. While chemists have previously exploited Δ*H* to direct the formation of desired constructs, these findings reveal that Δ*S* ultimately drives the synthetic process of multi-component self-assembly.

### MD modeling of arbitrary M_*n*L*n*_ and M_*n*L*n*+1_ assemblies

**Fig. 4:**
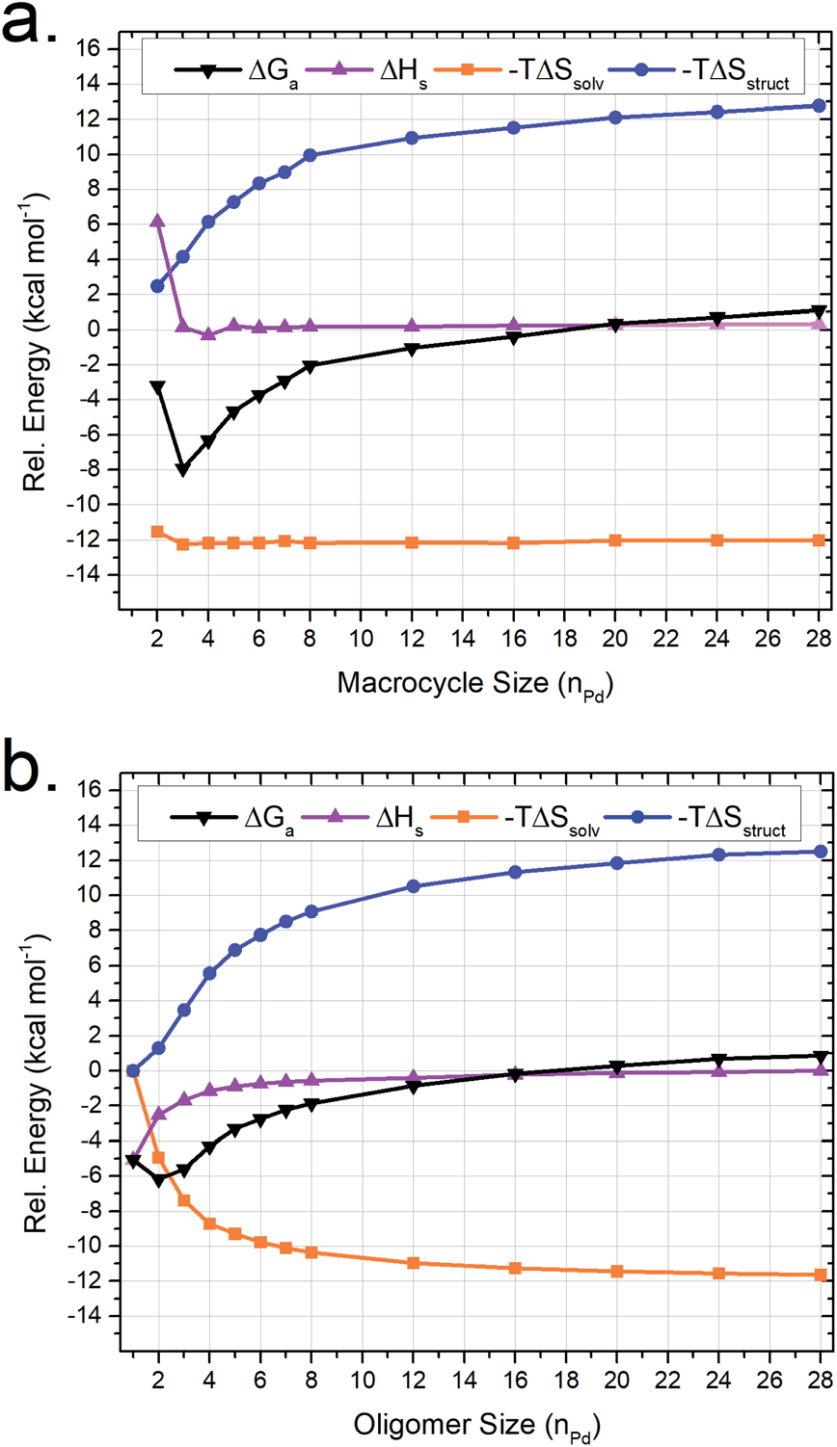
MD-calculated thermodynamic parameters for (a) M_*n*_^lin^L_*n*_ macrocyclic and (b) M_*n*_^lin^L_*n*+1_ oligomeric assemblies. Energy values are plotted on a common scale where −*T*Δ*S*_solv_ and −*T*Δ*S*_struct_ contributions are plotted relative to M_1_^lin^L_2_ while Δ*H* is plotted relative to M_28_^lin^L_29_. The sum of −*T*Δ*S*_solv_, −*T*Δ*S*_struct_ and Δ*H* values is plotted as Δ*G*.

Oligomers similar to (and including) M_2_^lin^L_3_ are theorized to form as intermediates in the self-assembly process of larger supramolecular structures (Scheme 1). Therefore, we utilized our MD-based approach to compare a range of oligomeric intermediates (M_*n*_^lin^L_*n*+1_; *n* = 1–28) and the potential macrocyclic products (M_*n*_^lin^L_*n*_; *n* = 2–28) to elucidate the role of individual thermodynamic parameters (Δ*S*_solv_, Δ*S*_struct_, and Δ*H*) on Δ*G* for the self-assembly of macrocycles (Fig. 4).

The resulting simulations reveal that both macrocyclic (Fig. 4a) and oligomeric (Fig. 4b) assemblies exhibit increasingly unfavorable Δ*G* with increasing assembly size driven by Δ*S*_struct_ contributions. The limited range of assemblies observed by NMR measurements (*i.e.*, M_2_^lin^L_3_, M_3_^lin^L_3_, and M_4_^lin^L_4_) is rationalized by the elevated Δ*G* experienced for other possible structures. This outcome is consistent with ESI-HRMS analysis (Fig. S9†) that provides qualitative evidence for the existence of larger assemblies in low abundance (*i.e.*, low signal-to-noise).

The value of −*T*Δ*S*_struct_ increases with size (Fig. 4, blue trace) for the self-assembly of both oligomeric and macrocyclic products, consistent with the decreased degrees-of-freedom experienced upon aggregation.^57^ Intriguingly, we find a non-linear correlation between the size and Δ*H* of oligomeric assemblies that is absent for macrocyclic congeners. Visualization of MD trajectory data reveals that larger oligomer assemblies adopt a compact conformation (Fig. S10†), resulting in increased strain (*i.e.*, Δ*H* penalty) on the palladium–pyridyl bonds compared to the zig-zag conformation found in smaller assemblies such as M_2_^lin^L_3_ (Fig. 3c). We infer that these compact suprastructures are necessary to realize a more compact assembly, akin to the folded structure observed for M_4_^lin^L_4_ macrocycles (Fig. 3b). This trade-off between −*T*Δ*S*_solv_ and Δ*H* originates from solvation and distinguishes oligomeric assemblies from macrocyclic ones. As −*T*Δ*S*_solv_ favors the formation of compact suprastructures, it is reasonable to deduce that the self-assembly of product macrocycles, in general, is driven by Δ*S*_solv_ contributions.

### MD modeling of reticular cage self-assembly

**Fig. 5:**
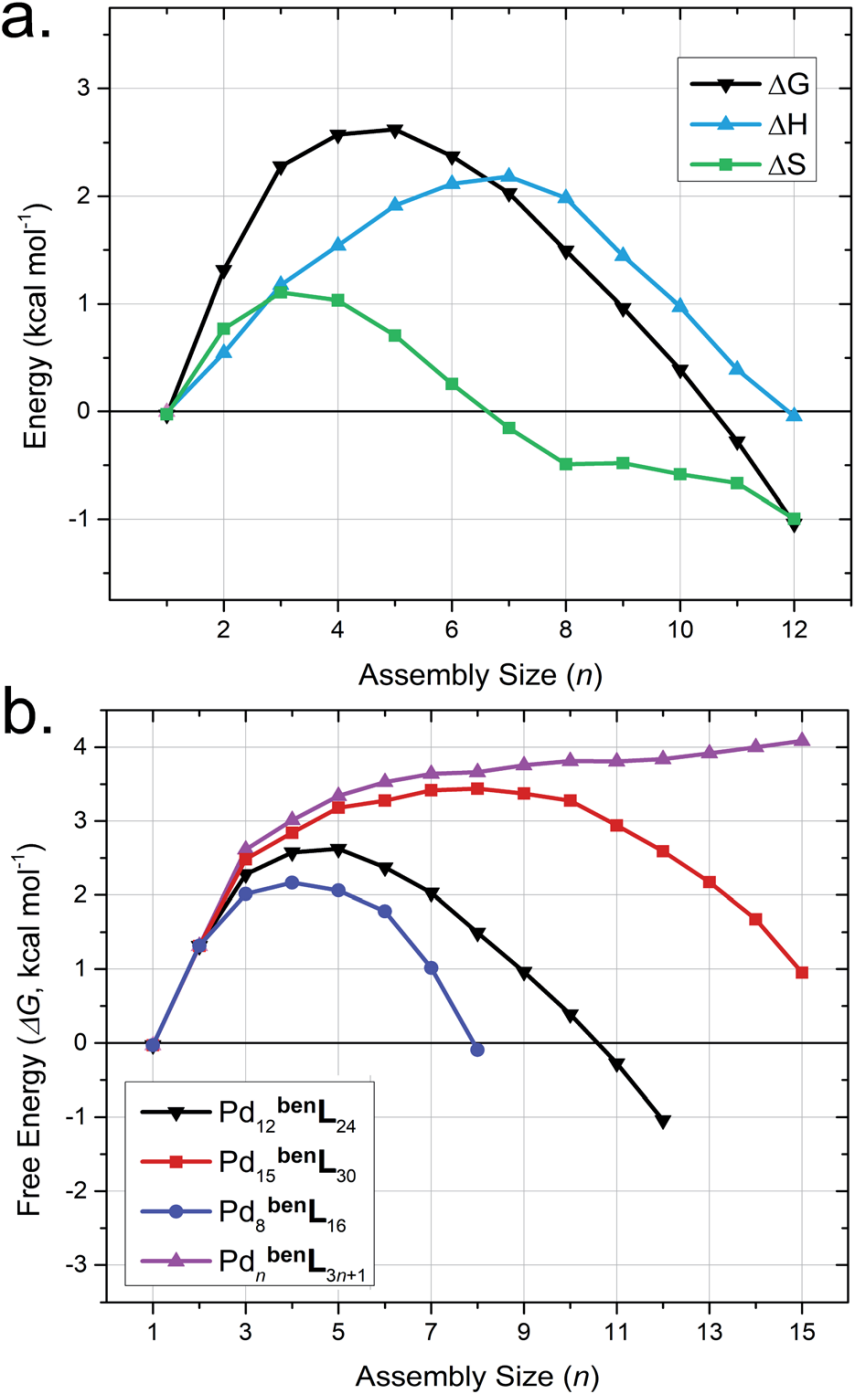
MD-calculated thermodynamic parameters for the Pd_*n*_^ben^L_*m*_ reticular intermediates (2*n* ≤ *m* ≤ 3*n*) for the self-assembly of Pd_12_^ben^L_24_ cages (a), alongside a comparison of the Δ*G* for Pd_*n*_^ben^L_*m*_ reticular intermediates (2*n* ≤ *m* ≤ 3*n* + 1) for the self-assembly of Pd_12_^ben^L_24_, Pd_15_^ben^L_30_, Pd_8_^ben^L_16_ cages and Pd_n_^ben^L_3n+1_ polymers (b). Computed thermodynamic parameters values are presented relative to the Pd_1_^ben^L_4_ complexes.

Mixtures of bent ditopic ligands (^ben^L = 4,4'-(5-ethoxy-1,3-phenylene)dipyridine) and free palladium(ii) coordination nodes (Pd) self-assemble to afford cuboctahedral cages (Pd_12_^ben^L_24_) *via* oligomeric-assembly intermediates. We employed our MD-based approach to gain insight into the self-assembly process,^52^ as incomplete or partial cages (*i.e.*, intermediate assemblies) are currently inaccessible by experimental methods (*e.g.*, CSI-HRMS, ^1^H-NMR).^51^ We developed models for intermediates of self-assembly (Pd_*n*_^ben^L_*m*_, where 2*n* ≤ *m* ≤ 3*n* + 1) from contiguous sections extracted from Pd_12_^ben^L_24_, Pd_8_^ben^L_16_, and Pd_15_^ben^L_30_ cages, as well as a polymeric state (Pd_*n*_^ben^L_*m*_, where *m* = 3*n* +1) geometries. Then we elucidated the self-assembly pathway for these partially-formed constructs from their respective Δ*S*, Δ*H*, and Δ*G* values from MD-simulation (Fig. 5).

Our simulations reveal the Pd_12_^ben^L_24_ exhibits a lower Δ*H* (*i.e.*, minimal geometric strain) compared to congeneric assemblies (Table 4). This observation is consistent with the literature and originates from the decreased metal–ligand bond strain experienced by this particular assembly-configuration.^8–13,49,52^ Models of partially-formed assemblies (*e.g.*, Pd_5_^ben^L_14_) bear an elevated Δ*H* (Fig. 5a, blue trace) as a result of strain originating from deflation or collapse during MD simulations (Fig. S11†). Parallel observations have been made in M_4_^lin^L_4_ macrocycles (Table 2), inferring increases in strain can act to offset penalties from solvation entropy (*i.e.*, Δ*S*_solv_), which leads to an overall elevation in Δ*H* for the system. The sum of entropic contributions (*i.e.*, Δ*S*_struct_ + Δ*S*_solv_ = Δ*S*, Fig. 5a, green trace) suggests that the formation of early intermediates (*n* <7) is hindered while the self-assembly of spherical cages (*n* = 12) is encouraged. These results demonstrate that while Δ*H* directs the polyhedral geometry of the final assembly,^52,55^ Δ*S* drives the structure of self-assembly to be spherical.

**Table 4:** MD-trajectory derived thermodynamic parameters of Pd_*n*_^ben^L_2*n*_ cages and Pd_*n*_^ben^L_3*n*+1_ polymers

Assembly	Thermodynamic parameters (kcal mol^−1^)^a^			
	Δ*H*°	−*T*Δ*S*_struct_°	−*T*Δ*S*_solv_°	Δ*G*°
Pd_8_^ben^L_16_	+1.20	+6.58	−7.88	−0.10
Pd_12_^ben^L_24_	−0.04	+6.90	−7.90	−1.04
Pd_15_^ben^L_30_	+1.70	+7.02	−7.77	+0.95
Pd_15_^ben^L_46_^b^	+1.60	+5.89	−3.41	+4.08

a Thermodynamic values are relative to those computed for Pd_1_^ben^L_4_ assemblies.

b Linear polymer assembly with the composition Pd_*n*_^ben^L_3*n*+1_.

The comparison of the free energy (Δ*G*) pathways for the self-assembly of different topologies (Fig. 5b) enables us to distinguish between the thermodynamic product (*i.e.*, Pd_12_^ben^L_24_), kinetic traps (*e.g.*, Pd_8_^ben^L_16_),^55^ and unrealized topologies (*e.g.*, Pd_15_^ben^L_30_, red trace). The maximum Δ*G* of intermediate assemblies of the Pd_15_^ben^L_30_ pathway (Δ*G*_max_ = +3.44 kcal mol^−1^) is greater than that of the Pd_12_^ben^L_24_ cage (Δ*G*_max_ = +2.62 kcal mol^−1^), rationalizing the possibility that intermediates may spontaneously reconfigure towards the latter structure as it is thermodynamically favored. In contrast, the Pd_8_^ben^L_16_ pathway features the lowest maximum energy (Δ*G*_max_ = +2.17 kcal mol^−1^) of analyzed pathways demonstrating that kinetic traps are readily identified by our MD-based approach.

## Conclusion

The thermochemical analysis of the self-assembly processes in palladium-based coordination macrocycles revealed unexpected Δ*S*-contributions that drive the formation of higher-order macrocycle assemblies (M_3_^lin^L_3_ and M_4_^lin^L_4_) from oligomer intermediates (M_2_^lin^L_3_). Using an MD-based approach, we found that the driving force for self-assembly originates from the solvation entropy (*i.e.*, Δ*S*_solv_) of oligomeric intermediates that effects surface-area minimization of the construct. Thermodynamic trends were established by MD analysis of larger assemblies, revealing that both Δ*S*_solv_ and Δ*S*_struct_ direct the formation of assemblies that exhibit similar Δ*H*. Data from MD models of formation pathways for palladium-based coordination cages reveal that Δ*S*_solv_ is responsible for driving the self-assembly process. Further application of our MD approach enables rationalization of the formation Pd_12_^ben^L_24_ cage products over kinetically trapped congeners (*i.e.*, Pd_8_^ben^L_16_) directly from the computed thermodynamic quantities (Δ*S*_solv_, Δ*S*_struct,_ and Δ*H*) of the intermediate assemblies. Overall, these complementary experimental and computational investigations expose Δ*S* as the driver for the formation of these desirable highly ordered structures that have broad applications across supramolecular chemistry.

## Author contributions

DAP conceived, designed and performed the experiments. EOB contributed ligand materials, performed HR-MS analyses and provided experimental expertise for self-assembly experiments. JNHR and SM supervised the work. DAP, SM, and JNHR wrote the manuscript with all authors providing significant contributions to the analysis and interpretation of the work.

## Conflicts of interest

There are no conflicts to declare.

## Supplementary Material

SC-013-D2SC03154J-s001
