# Supplementary material for: Entropy directs the self-assembly of supramolecular palladium coordination macrocycles and cages
Source: Chem Sci. 2022 Aug 10;13(34):10141–8. doi: 10.1039/d2sc03154j (PMC9430592; doi:10.1039/d2sc03154j)
Supplement: SC-013-D2SC03154J-s001 [file SC-013-D2SC03154J-s001.pdf]

Electronic Supplementary Material (ESI) for Chemical Science.
This journal is © The Royal Society of Chemistry 2022

Supporting Information for

Entropy directs the self-assembly of supramolecular palladium coordination macrocycles and cages

D. A. Poole III,[a] E. O. Bobylev,[a] S. Mathew,[a] and J. N. H. Reek[a*]

Homogeneous, Supramolecular, and Bioinspired Catalysis group, van't Hoff Institute for Molecular Science (HIMS), University of Amsterdam (UvA), Science Park 904, 1098 XH Amsterdam, The Netherlands

J.N.H.Reek@uva.nl

Contents

1. Experimental ... 3
Scheme S1. ... 3
2. ^{1}H-NMR and DOSY characterization of $\mathbf{M}_n{}^{\mathbf{lin}}\mathbf{L}_n$ and $\mathbf{M}_n{}^{\mathbf{lin}}\mathbf{L}_{n+1}$ assemblies ... 4
Figure S1. ... 4
Figure S2. ... 4
Figure S3. ... 5
3. Molecular dynamics simulations ... 6
Figure S4. ... 6
4. Simulation length dependence for computed thermodynamic parameters ... 7
Figure S5 ... 7
Figure S6 ... 7
Figure S7 ... 8
5. Solvation-directed folding of $\mathbf{M}_4{}^{\mathbf{lin}}\mathbf{L}_4$ assemblies ... 9
Figure S8. ... 9
6. ESI–MS identification of oligomer assemblies ... 10
Figure S9 ... 10
7. Visualized helical suprastructures of $\mathbf{M}_n{}^{\mathbf{lin}}\mathbf{L}_{n+1}$ coordination polymers ... 11
Figure S10. ... 11
8. Conformational changes of unstable reticular polymer intermediates ... 11
Figure S11 ... 11
9. Thermochemical analysis procedure ... 12
10. VT-NMR data ... 13
Figure S12. ... 13
Figure S13. ... 14
Figure S14. ... 14
Figure S15. ... 15
Figure S16. ... 15
Table S1 ... 16
Figure S17 ... 18
Figure S18 ... 19
Figure S19 ... 19
11. Graphical explanation of ΔH and $-T\Delta S$ reference states ... 20
Figure S20 ... 20
12. References ... 21

1. Experimental

Solvents (Acetonitrile, Acetone), drying agents (sodium sulfate) and nitric acid (conc. 15.6 M) were purchased from VWR. Acetone was dried directly before use by vigorous stirring with sodium sulfate (Na_2SO_4, 10 g L^{-1}), which was removed by vacuum filtration (glass frit, porosity 4). Nitric acid (100 µL,15.6 M) was diluted with purified water (MilliQ, 18.1 mΩ) in a volumetric flask (100 mL). Deuterated DMSO (99.0 %D, d_6-DMSO) was purchased from Merck (SKU: 151874) and used as delivered.

Purchased reagents, dichloro(*N*,*N*,*N*',*N*'-tetramethylethylenediamine)palladium(II) (Merck, SKU: 345229), $AgNO_3$ (Fluorochem, SKU: 080282), and 4,4'-dipyridyl (TCI, B0469) were used as delivered.

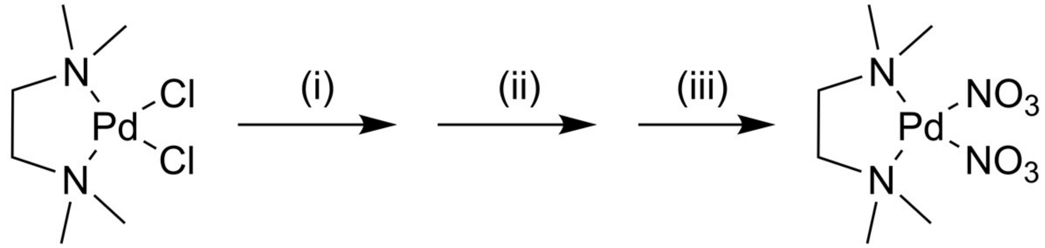

Scheme S1. Overview for the synthesis and purification of dinitrato(*N*,*N*,*N*′, *N*′-tetramethylethylenediamine)palladium(II) (**M**), from dichloro(*N*,*N*,*N*′, *N*′-tetramethylethylenediamine)palladium dichloride. Conditions: (i) 2.05 eq. $AgNO_3$, cat. HNO_3, MeCN/H_2O 1:1, dark, stir 72 h; (ii) filter to remote precipitated AgCl, dry; (iii) dissolve in acetone, filter to remove insoluble $AgNO_3$, dry.

M: The coordination node, dinitrato(*N*,*N*,*N*′,*N*′-tetramethylethylenediamine)palladium(II) (**M**), was synthesized and purified following a modified literature procedure.[1] Briefly, dichloro(*N*,*N*,*N*′,*N*′-tetramethylethylenediamine)palladium(II) (1.00 g, 3.4 mmol) was suspended in acetonitrile (100 mL) in a 250 mL foil-wrapped amber round-bottom flask and stirred until a clear yellow solution was obtained. Then, a solution of $AgNO_3$ (1.19 g, 7.0 mM) in dilute aqueous nitric acid (1.5 mM, 100 mL), was added by an ordinary funnel, resulting in the immediate formation of a white precipitate (AgCl). The flask was closed by glass stopper and left stirring under ambient conditions (ca. 72 h). The reaction mixture was then filtered by gravity to remove precipitated AgCl (Whatman Grade 313), and the filtrate dried to afford an off-yellow residue. This residue was suspended in dried acetone (500 mL) and vigorously stirred under a dry nitrogen atmosphere (2 h). The resulting yellow solution was filtered (0.44 µm), and dried to afford (1.01 g, 86% yield) the title compound as a yellow crystalline powder. ^{1}H-NMR (300 MHz, d_6-DMSO) δ 2.55 (6 H), 2.80 (2 H).

2. ^{1}H-NMR and DOSY characterization of $\mathbf{M}_n{}^{\mathbf{lin}}\mathbf{L}_n$ and $\mathbf{M}_n{}^{\mathbf{lin}}\mathbf{L}_{n+1}$ assemblies

^{1}H-NMR spectra were measured on a Bruker DRX 300 (^{1}H freq. = 300 MHz) equipped with a variable temperature controller and high-resolution pulsed z-gradient probe. Spectral data were referenced to internal TMS standard (0.00 ppm).

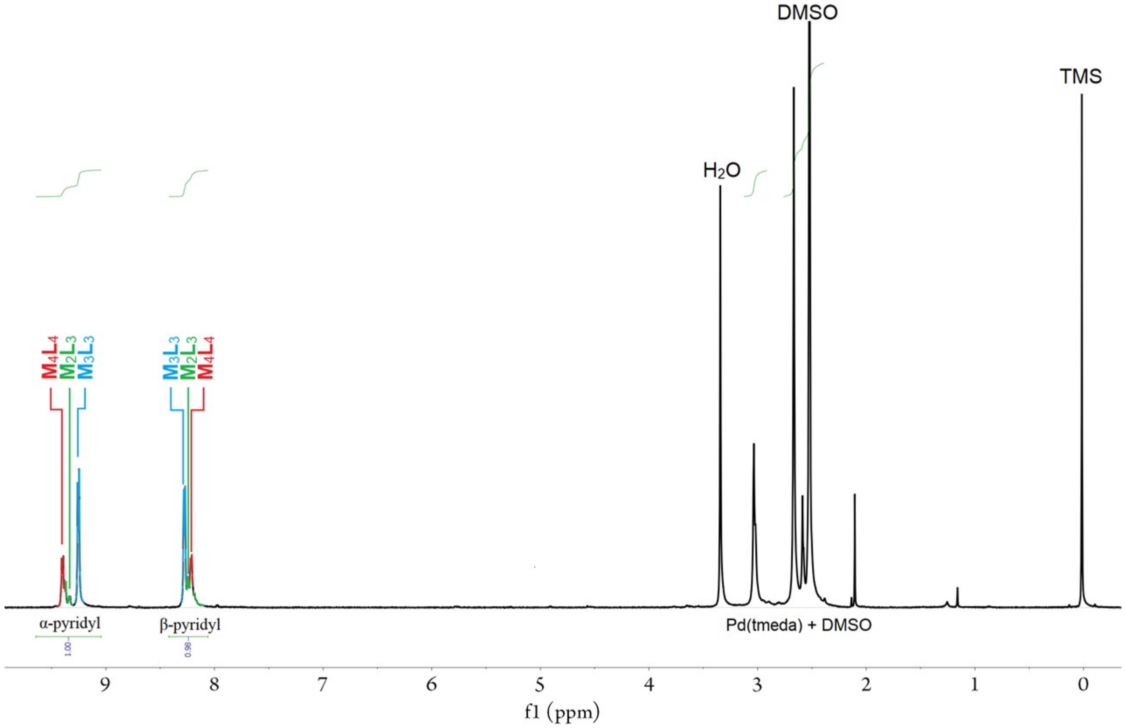

Figure S1. ^{1}H-NMR spectra (300 MHz) of assemblies ($\mathbf{M}_2{}^{\mathbf{lin}}\mathbf{L}_3$, $\mathbf{M}_3{}^{\mathbf{lin}}\mathbf{L}_3$, $\mathbf{M}_4{}^{\mathbf{lin}}\mathbf{L}_4$) in d_6-DMSO at 298 K.

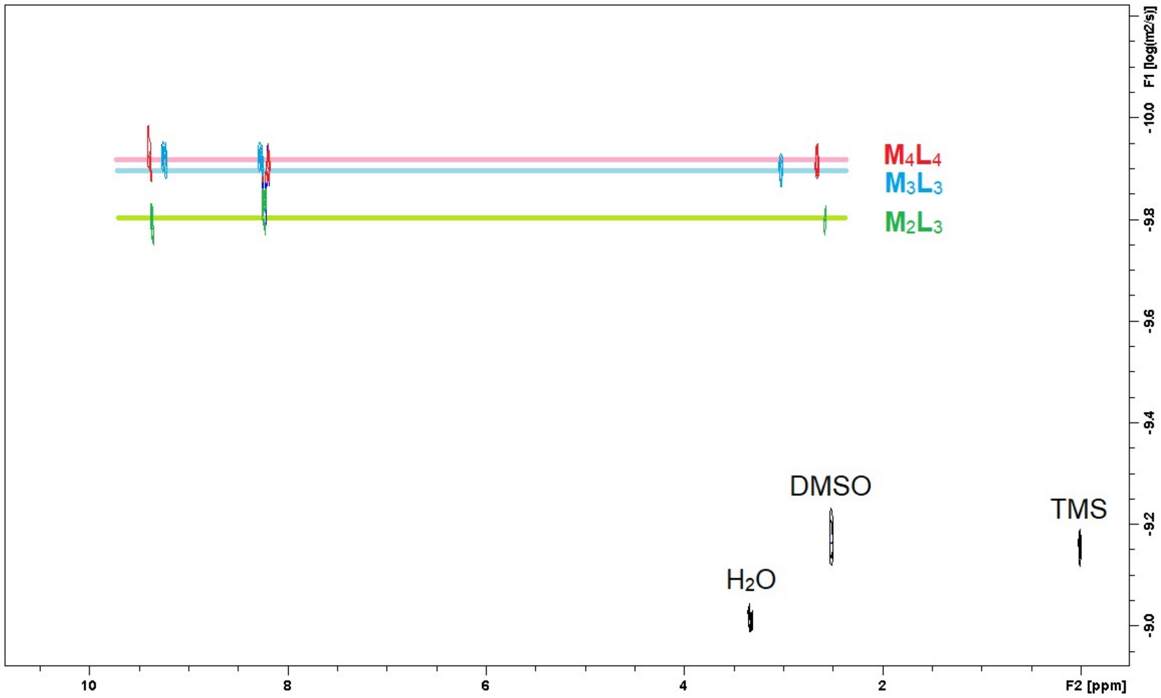

Figure S2. Diffusiogram of coordination assemblies ($\mathbf{M}_2{}^{\mathbf{lin}}\mathbf{L}_3$, $\mathbf{M}_3{}^{\mathbf{lin}}\mathbf{L}_3$, $\mathbf{M}_4{}^{\mathbf{lin}}\mathbf{L}_4$) computed from the variable gradient pulse sequence (300 MHz, d_6-DMSO, 298 K, Δ_t = 100 ms, δ = 1 ms, 19 points, ledbpgp2s) using the dosy2d processing program in Topspin 4.0.9.

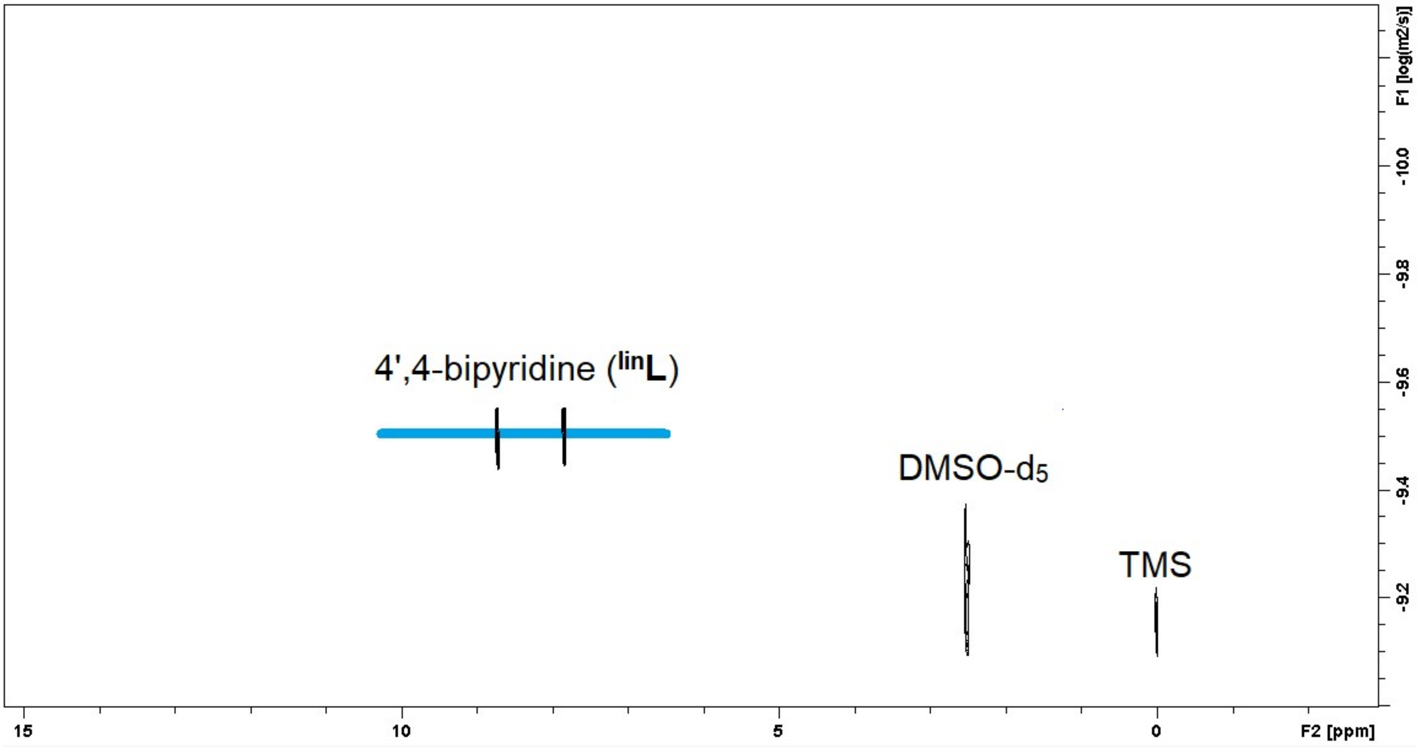

Figure S3. Diffusiogram of free 4,4'-bipyridine (^{lin}**L**) computed from the variable gradient pulse sequence (300 MHz, d_6-DMSO, 298 K, Δ_t = 100 ms, δ = 1 ms, 19 points, ledbpgp2s) using the dosy2d processing program in Topspin 4.0.9.

3. Molecular dynamics simulations

Parameters were optimized for Pd–(tmeda) and Pd(tmeda)–4,4'-bipyridyl interactions following a previously reported protocol.[2] Quantum mechanical molecular dynamics trajectories were obtained for a model $\mathbf{M}_2{}^{lin}\boldsymbol{L}_3$ oligomer using *xtb* (GFN2-xTB),[3] which were subsequently used for single point DFT calculations using *Gaussian16 Rev C.* at a b3lyp/def2tzv theory level.[4] Parameters were then optimized using *paramfit* to obtain a high-fidelity reproduction of the DFT energies (Figure S3).[5]

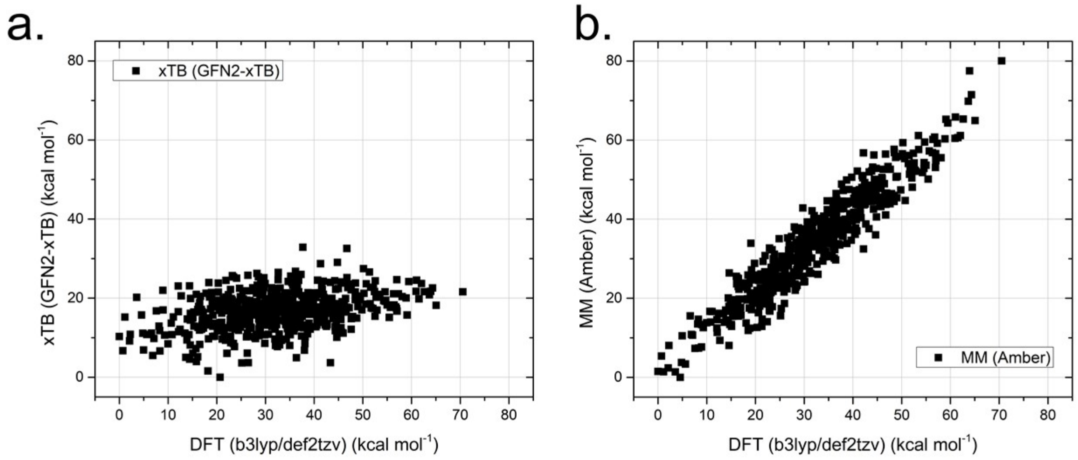

Figure S4. Comparison of DFT single point energies for configurations of the $\mathbf{M}_2{}^{\mathbf{lin}}\mathbf{L}_3$ oligomer to energies obtained from (a) semi-empirical (GFN2-xTB) and (b) molecular-mechanical (optimized Amber forcefield). Frames were generated from a molecular dynamics simulation using xtb ($n_{configuraiton} = 512$).

Models of oligomer ($\mathbf{M}_n{}^{\mathbf{lin}}\mathbf{L}_{n+1}$) and macrocycle ($\mathbf{M}_n{}^{\mathbf{lin}}\mathbf{L}_n$) assemblies were prepared using tleap, included in the Ambertools17 software suite.[6] Molecular dynamics were carried out using GPU-accelerated *pmemd.cuda*[7] included in the Amber16 suite. Trajectories were analyzed with *cpptraj* to obtain thermodynamic values, detailed in Equations S1–3.[8]

$$\Delta H = \frac{\Delta U_{FF(M+L)} - \left(\Delta U_{FF(M)} + \Delta U_{FF(L)}\right)}{n_{Pd}} \qquad (Eq.S1)$$

Equation S1. The enthalpy (ΔH) of assemblies featuring n palladium coordination nodes (n_{Pd}) was computed subtraction of the forcefield energy of the whole assembly ($\Delta U_{FF(M+L)}$) and its constituent components ($\mathbf{M} = \Delta U_{FF(M)}$, ${}^{\mathbf{lin}}\mathbf{L} = \Delta U_{FF(L)}$).

$$\Delta S_{struct} = \frac{\Delta S_M(n)}{n_{Pd}} - \Delta S_M(1) \qquad (Eq.S2)$$

Equation S2. The structural entropy (ΔS_{struct}) of assemblies featuring n palladium coordination nodes (n_{Pd}) was computed as the difference between the average entropy of an oligomer of size n ($\Delta S_M(n)$) (Equation S2, minuend), and the entropy of $\mathbf{M}_1{}^{\mathbf{lin}}\mathbf{L}_2$ (Equation S2, subtrahend). Entropies were computed by quasi-harmonic analysis of palladium-centers, excluding contributions of the lighter atoms.[9]

$$\Delta S_{solv} = \frac{\Delta S_{gbsa}}{n_{Pd}} \qquad (Eq.S3)$$

Equation S3. The solvation entropy (ΔS_{solv}) of assemblies featuring n palladium coordination nodes (n_{Pd}) was computed as the non-polar solvation contribution of the implicit solvation model (ΔS_{gbsa}) scaled by the number of coordination nodes (n_{Pd}).

4. Simulation length dependence for computed thermodynamic parameters

Accurate determination of thermodynamic parameters ($-T\Delta S_{struct}$, $-T\Delta S_{solv}$, and ΔH) requires sufficient sampling of configuration space. The optimal length was determined by sampling a long (100 ns) MD trajectory of the largest assembly (**M_{28}**$^{\mathbf{lin}}$**L_{29}**) at various points to minimize error below 5% of the asymptotic value.

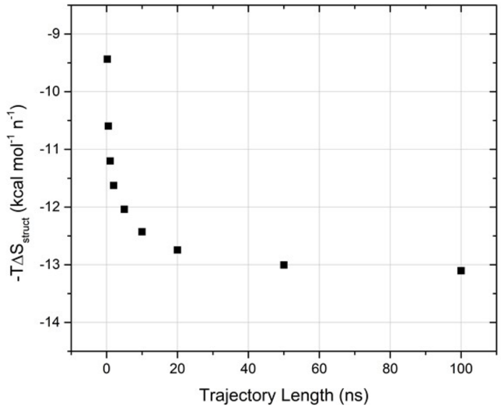

Figure S5. Structural entropy ($-T\Delta S_{struct}$, Equation S2) determined for different trajectory lengths (1–100 ns).

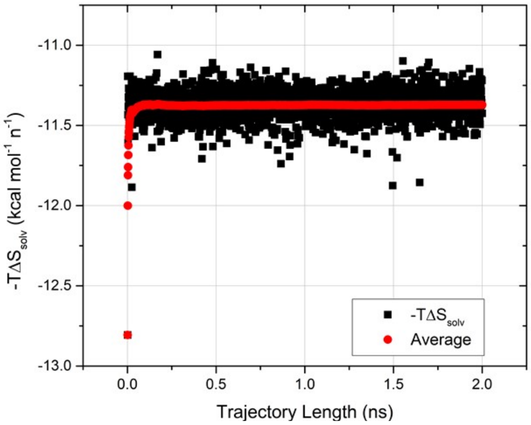

Figure S6. Solvation entropy (Equation S3) determined for different trajectory lengths (0–2 ns). Energies at sampled trajectory frames are shown as black squares, with a running average provided in red circles.

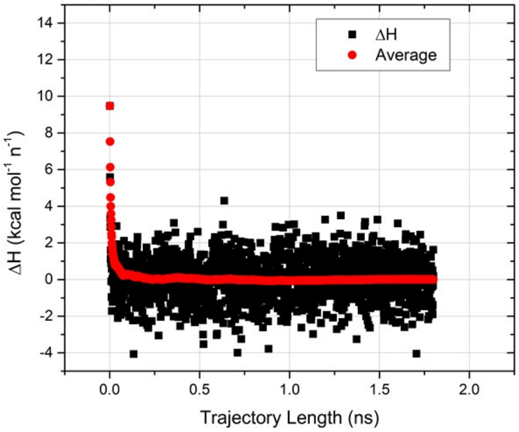

Figure S7. Enthalpy (Equation S1) determined for different trajectory lengths (0–2 ns). Energies at sampled trajectory frames are shown as black squares, with a running average provided in red circles.

5. Solvation-directed folding of $\mathbf{M_4^{lin}L_4}$ assemblies

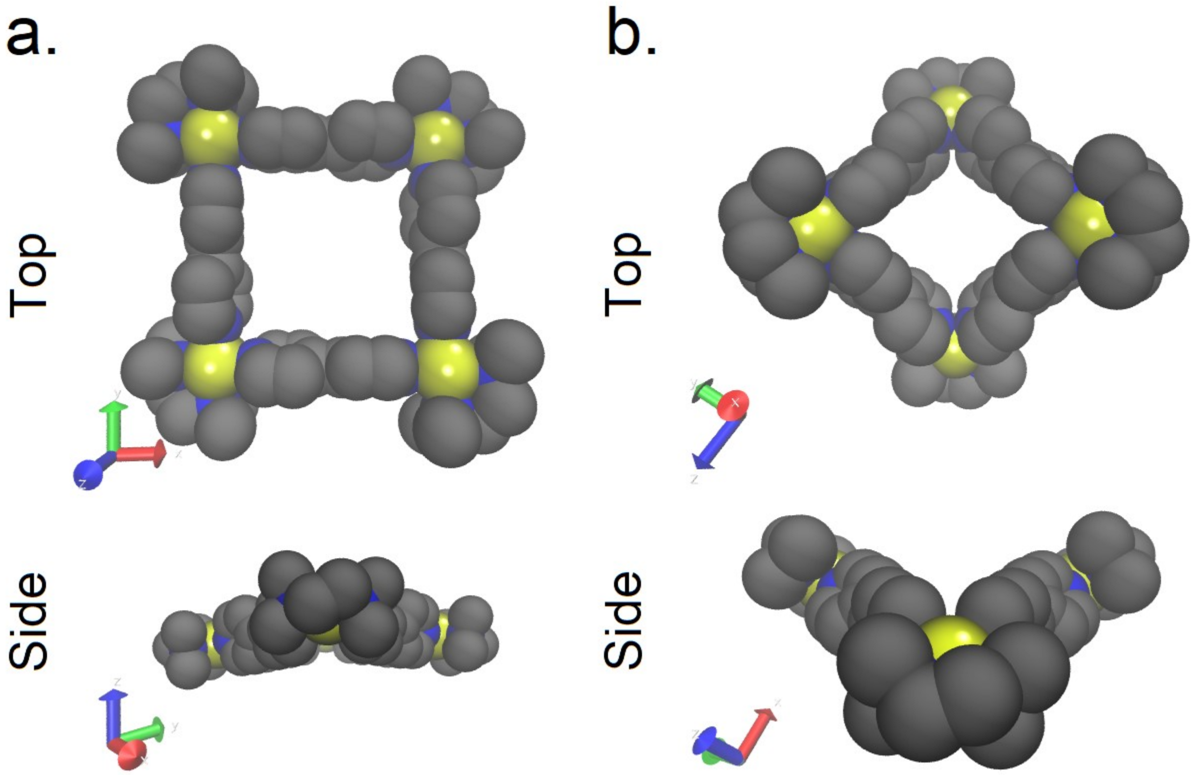

Figure S8. Orthogonal (top and side) projections of van der Waals renderings of MD-optimized $\mathbf{M_4^{lin}L_4}$ assemblies without (a) and with (b) implicit solvation. Hydrogen atoms are omitted for clarity.

To examine the structural dynamics of $\mathbf{M_4^{lin}L_4}$, additional MD simulations were to compare the $\angle_{N\text{–}Pd\text{–}N}$ for structures with GBSA solvation and those simulated with vacuum conditions. While simulations featuring GBSA solvation featured a hyperbolic geometry (Figure S8b, $\angle_{N\text{–}Pd\text{–}N} = 85°$), under vacuum conditions the system came closer to the ideal angles for square-planar coordination ($\angle_{N\text{–}Pd\text{–}N} = 88°$).

6. ESI–MS identification of oligomer assemblies

Electrospray ionization mass spectrometry (ESI–MS): Mass spectra were collected on a HR-ToF Bruker Daltonik GmbH (Bremen, Germany) Impact II, an ESI-ToF MS capable of resolution of at least 40000 FWHM, which was coupled to a Bruker cryospray unit. Detection was in positive-ion mode and the source voltage was between 4 and 6 kV. The sample was introduced with a syringe pump at a flow rate of 18 µl hr^{-1}. The drying gas (N_2) was held at 40°C and the spray gas was held at 60°C. The machine was calibrated prior to every experiment via direct infusion of a Na(TFA) solution, which provided a *m/z* range of singly charged peaks up to 3500 Da. in both ion modes. Software acquisition using Compass 2.0 for OTOF series with further processing by *m-mass* for simulation data.

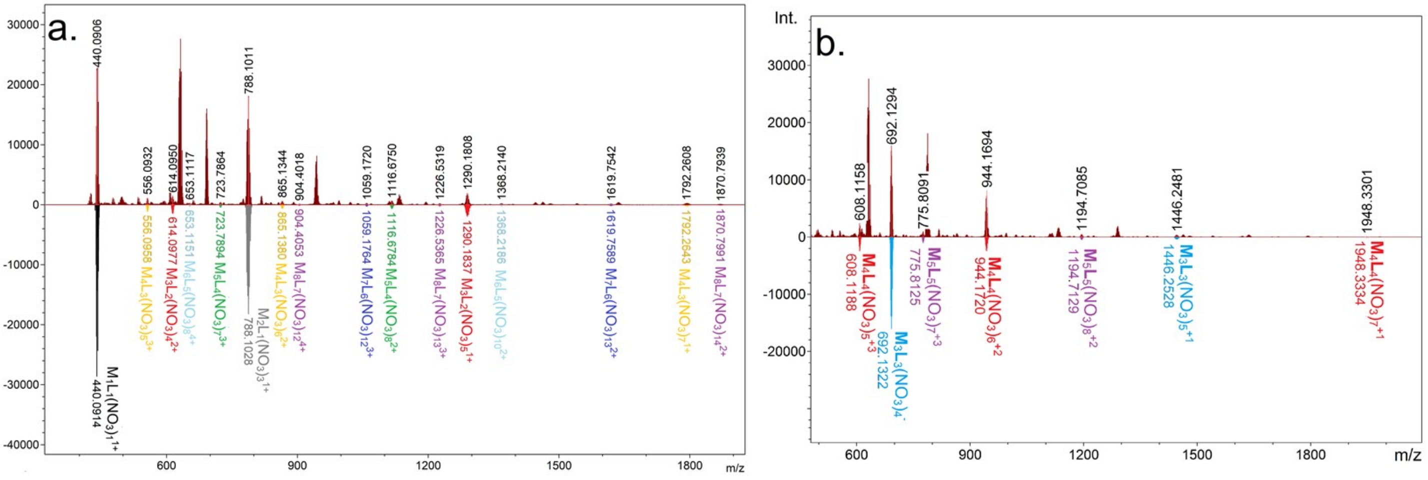

Figure S9. (a) ESI–MS spectra identifying oligomer assemblies with a composition of $\mathbf{M}_n{}^{\mathbf{lin}}\mathbf{L}_{n-1}$ (n = 2–8) in addition to a $\mathbf{M}_1{}^{\mathbf{lin}}\mathbf{L}_1$ complex, highlighted in various colors corresponding to the composition of **M** and $^{\mathbf{lin}}\mathbf{L}$. For readability, $^{\mathbf{lin}}\mathbf{L}$ is shown as **L**. Oligomer assemblies were identified by two ion peaks featuring a different number of associated counterions (NO_3^-) as labeled. (b) The same ESI–MS spectra but with presumed macrocycle assemblies with a composition of $\mathbf{M}_n{}^{\mathbf{lin}}\mathbf{L}_n$ (n = 3–5) identified.

In our ESI–HRMS spectra we observed a range of $\mathbf{M}_n{}^{\mathbf{lin}}\mathbf{L}_{n-1}$ species, mostly at low intensities. Weilandt et al. demonstrates with similar mono-nuclear complexes, the second of two attached ligands are significantly more liable ($\Delta\Delta G$ = 4.7 – 11.1 kcal mol^{-1}), especially in the gas-phase where entropic effects are diminished.[11] From this, we infer that peaks for $\mathbf{M}_n{}^{\mathbf{lin}}\mathbf{L}_{n-1}$ may arise as decomposition products from $\mathbf{M}_n{}^{\mathbf{lin}}\mathbf{L}_{n+1}$ by the loss of the outermost ligands. Specifically, the intense peak for $\mathbf{M}_2{}^{\mathbf{lin}}\mathbf{L}_1$ (*m/z* = 788.1028, Figure S9, gray) may arise as the *in situ* decomposition product of the NMR-observed $\mathbf{M}_2{}^{\mathbf{lin}}\mathbf{L}_3$ assembly, providing further support for its presence in solution.

7. Visualized helical suprastructures of $\mathbf{M}_n{}^{\mathbf{lin}}\mathbf{L}_{n+1}$ coordination polymers

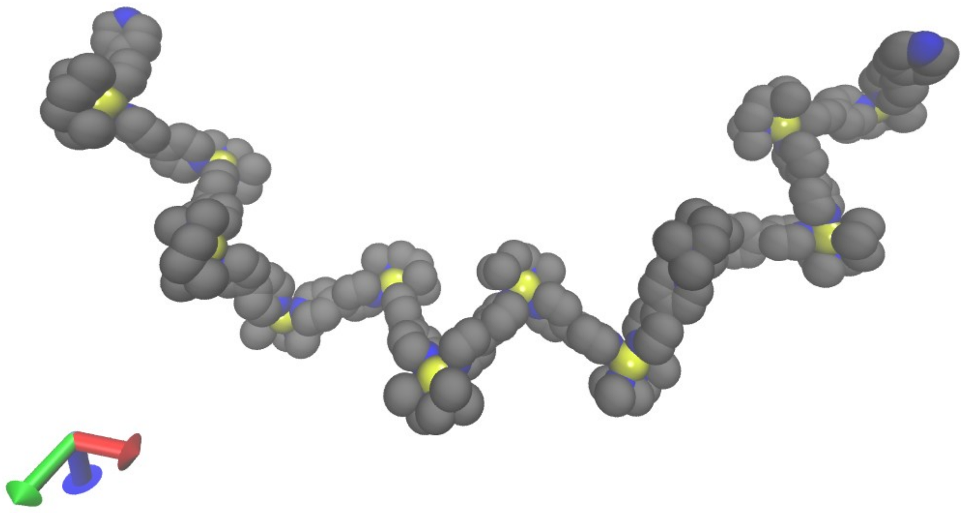

Figure S10. Visualization of MD trajectory snapshot of $\mathbf{M}_{12}{}^{\mathbf{lin}}\mathbf{L}_{13}$ oligomer compacted by solvation effects to adopt a helical suprastructure. Hydrogens omitted for clarity. In these larger oligomeric assemblies *N–Pd–N* angles are typically < 89°, leading to an elevated Δ*H* from strain.

8. Conformational changes of unstable reticular polymer intermediates

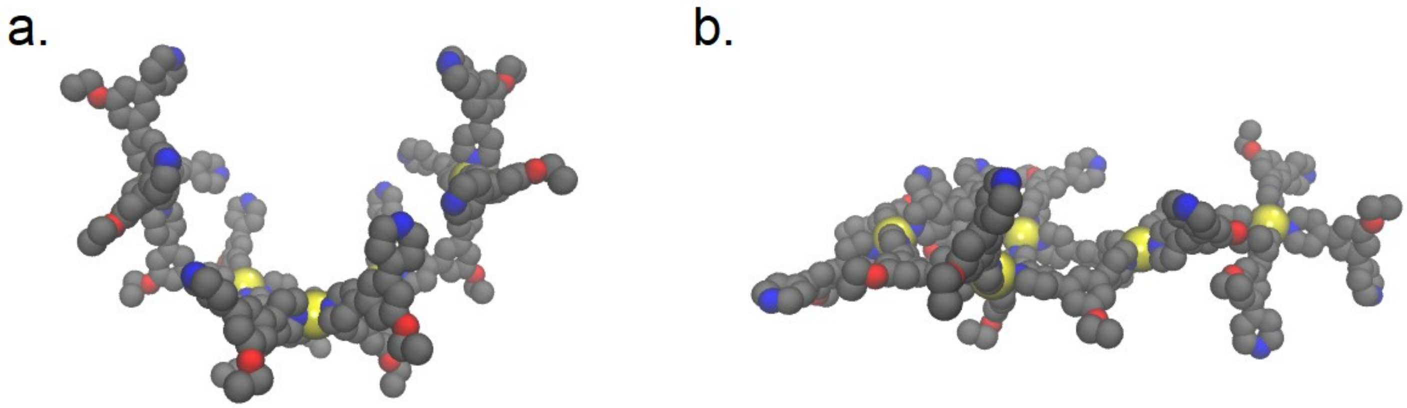

Figure S11. Visualization of MD trajectory snapshots of $Pd_5{}^{\mathbf{ben}}\mathbf{L}_{15}$ reticular intermediates as constructed with a $Pd_{12}{}^{\mathbf{ben}}\mathbf{L}_{24}$ template (a) and after 50 ns of molecular dynamics simulations (b). Hydrogens are omitted from clarity. While simulations begin with an initial curvature of the completed cage bearing a minimum Δ*H* (Figure 5a, blue trace), solvation forces minimize the overall curvature of the structure leading to a smaller solvent exposed surface area (i.e., -*T*Δ*S*, green trace).

9. Thermochemical analysis procedure

VT-NMR data (Figures S13 – S16) were obtained and regions corresponding to the α-pyridyl protons of $\mathbf{M_3^{lin}L_3}$, $\mathbf{M_4^{lin}L_4}$, and $\mathbf{M_2^{lin}L_3}$ assemblies were integrated (see Int. Regions and corresponding highlights, Figure S12). These were processed to obtain a *ΔG* value (Figure 2), which were thereafter fit with a modified van 't Hoff model to obtain *ΔS* and *ΔH*, shown a flow chart in Scheme S2.

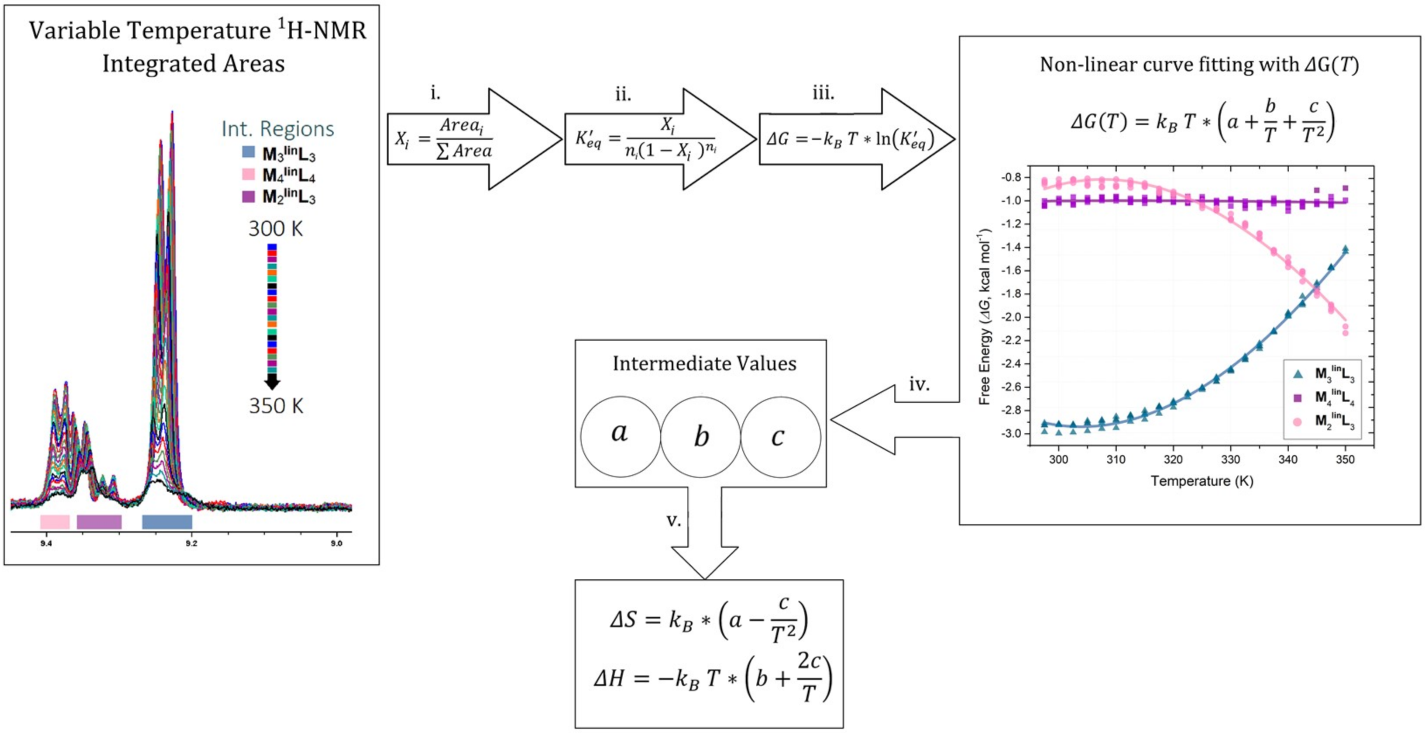

Scheme S2. Data processing scheme used to convert VT-NMR data into *ΔG* (i – iii), followed by non-linear curve-fitting to obtain *ΔS*, *ΔH* (iv, v). (i) The area data were converted to relative areas (X_i) by normalization. (i) An equilibrium constant K_{eq}' was computed for each assembly from its relative concentration assuming a common pool of reactants for assembly formation, these are equivalent to a partition function for the assemblies. (iii) The K_{eq}' values were converted to free energies using the Boltzmann equation. (iv) The *ΔG* values as a function of temperature were directly fit with an expanded van 't Hoff model.[10] (v) The values obtained from the fitting (*a*, *b*, *c*) were converted into *ΔS* and *ΔH*. While a single set of spectra is shown (left), all four sweeps were used to curve fitting (right). Individual steps are also illustrated below with the data (Figure S17).

We found that the peaks for all three assemblies decreased with elevating temperature in VT-NMR analysis (Figure S12). This attenuation was attributed to a more rapid relaxation occurring due to thermal motions, which we assume affected each assembly equally. Based on this assumption, the relative area (X_i) of the peaks reflects the quantity of $\mathbf{^{lin}L}$ distributed in $\mathbf{M_3^{lin}L_3}$, $\mathbf{M_4^{lin}L_4}$, and $\mathbf{M_2^{lin}L_3}$ assemblies (Scheme S2, i), and the relative concentration an assembly is $X_i \times n_i^{-1}$.

Based on the assumed mass balance, **M** is afforded alongside $\mathbf{M_2^{lin}L_3}$ (i.e., $\mathbf{M_3^{lin}L_3} \rightleftharpoons \mathbf{M_2^{lin}L_3}$+**M**). Thus, we simplify the system to treat $\mathbf{M_3^{lin}L_3}$, $\mathbf{M_4^{lin}L_4}$, and $\mathbf{M_2^{lin}L_3}$ as having the same composition, $\mathbf{M_n^{lin}L_n}$. We can then treat the system as an equilibrium for the formation of each assembly from a common pool of equimolar **M** and $\mathbf{^{lin}L}$ (i.e. $n\mathbf{M} + n\mathbf{^{lin}L} \rightleftharpoons M_nL_n$) providing an equilibrium constant, K_{eq}' (Scheme S2, ii).

Using this equilibrium constant with the Boltzmann equation (Scheme S2, iii) gives us a *ΔG* for each assembly, that reflects the relative energy difference between each assembly the reference state of a common pool of equimolar **M** and $\mathbf{^{lin}L}$. These *ΔG* values reflect only the thermodynamic differences between $\mathbf{M_3^{lin}L_3}$, $\mathbf{M_4^{lin}L_4}$, and $\mathbf{M_2^{lin}L_3}$, and is otherwise insensitive to (or robust against) the formation of un-observed NMR-silent assemblies.

Finally, direct non-linear fitting of these *ΔG* values as a function of temperature with expanded van 't Hoff equation (Scheme S2, iv),[10] provides relative *ΔS* and *ΔH* differences between $\mathbf{M_3^{lin}L_3}$, $\mathbf{M_4^{lin}L_4}$, and $\mathbf{M_2^{lin}L_3}$ (Scheme S2, v).

10. VT-NMR data

VT-NMR data were obtained between 297.5 and 350.0K in steps of 2.5 K with 5 minutes of equilibration followed by 1 minute of measurement with a recycle delay of 15 seconds. Four temperature sweeps were measured in total with spectra obtained along both trajectories. In addition, the final setpoint, 297.5 K, was measured twice as a quality control for the equilibration time used (Figure S19).

In general, we observe that the peaks corresponding to $\mathbf{M_3^{lin}L_3}$, $\mathbf{M_4^{lin}L_4}$, and $\mathbf{M_2^{lin}L_3}$ are attenuated by the increasing temperature while peaks for water and DMSO increase.

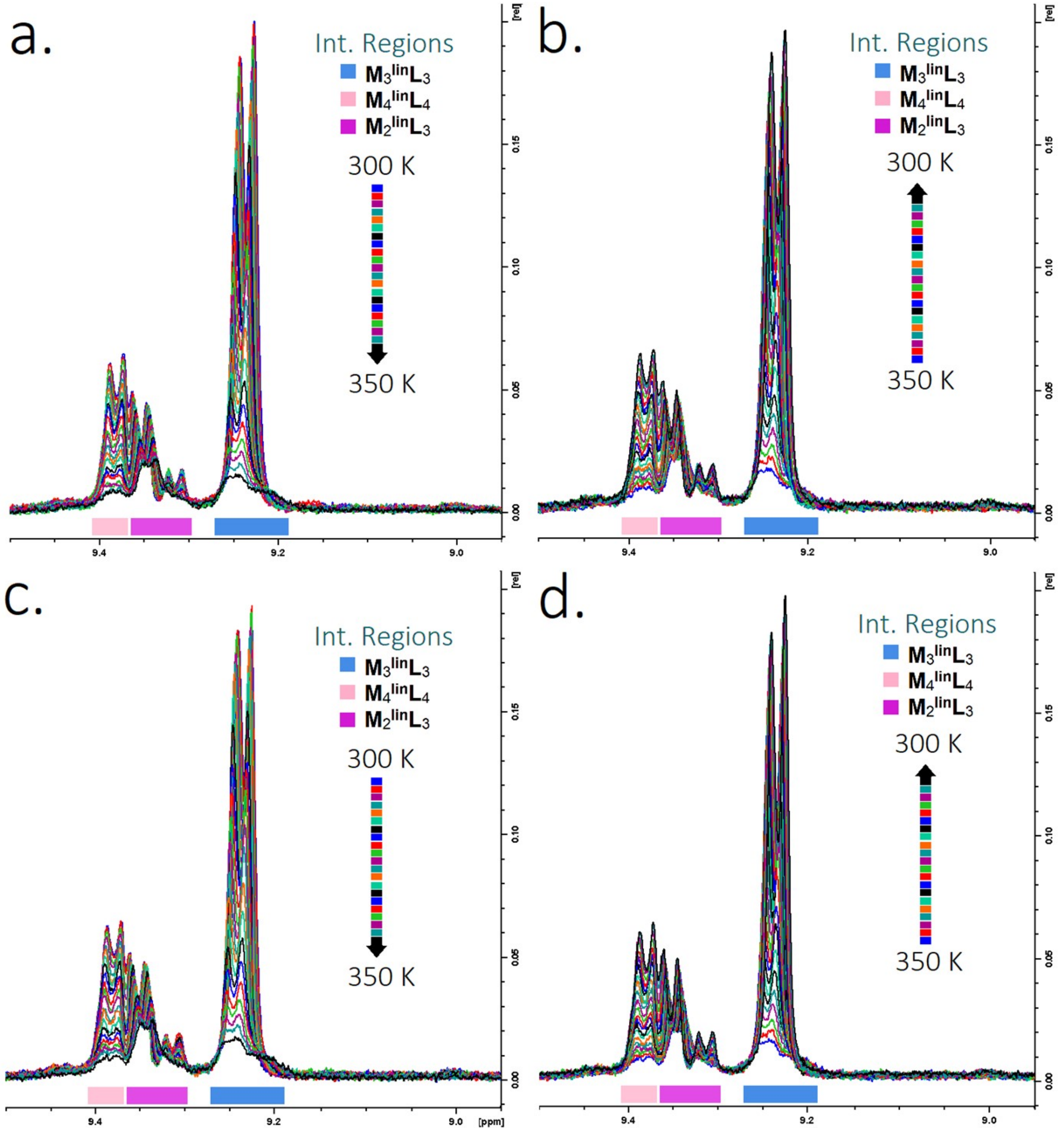

Figure S12. VT-NMR spectra obtained for the α-pyridyl region used to determine the distribution of $\mathbf{M_3^{lin}L_3}$, $\mathbf{M_4^{lin}L_4}$, and $\mathbf{M_2^{lin}L_3}$ with integration regions shown by a colored bar. Spectra are were measured consecutively but are here presented as separate heating or cooling segments: (a) 297.5 K → 350.0 K, (b) 350.0 K → 297.5 K, (c) 297.5 K → 350.0 K, (d) 350.0 K → 295.5 K. Complete spectra are provided below (Figures S13–S16) with a complete listing of integral results provided on Table S1. All ^{1}H-NMR spectra were obtained using a Bruker DRX 300 (^{1}H freq. = 300 MHz) with solvent d_6-DMSO.

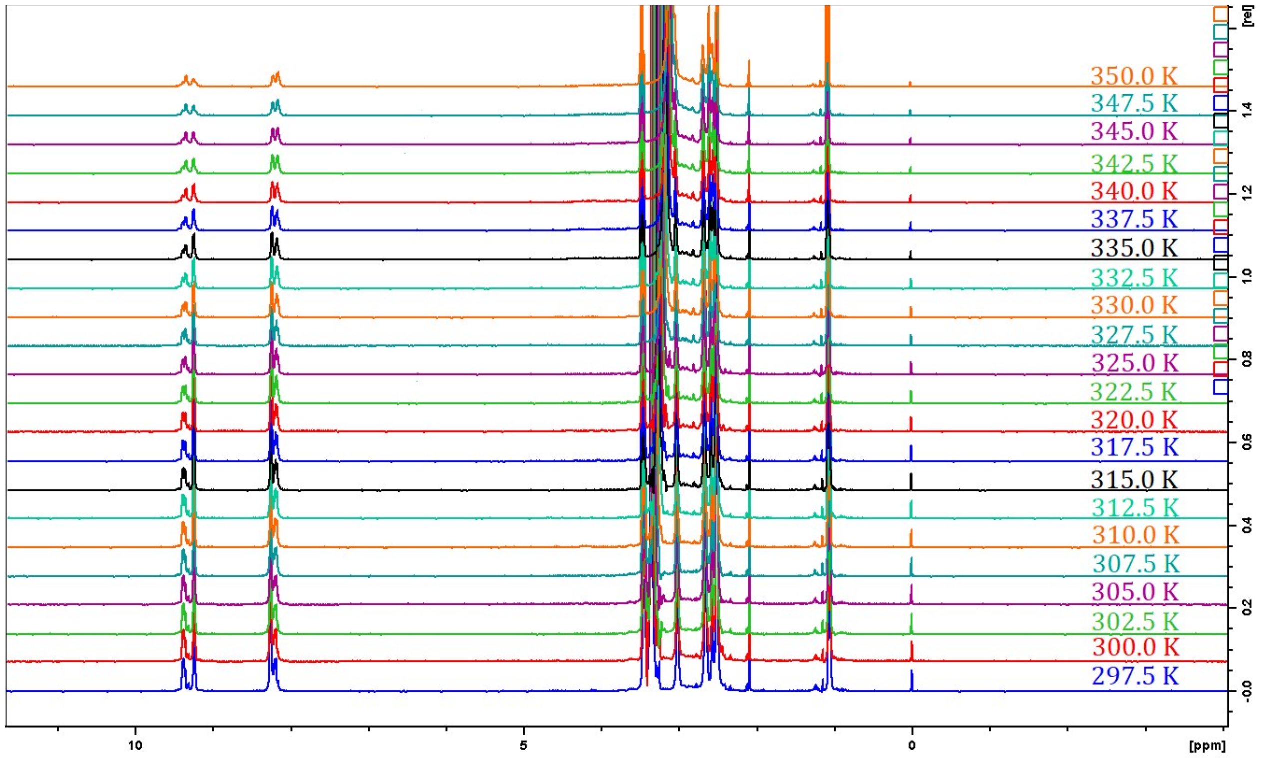

Figure S13. Full range spectra obtained over the first heating (297.5 K, bottom to 350.0 K top), with each spectra obtained at the listed temperature. A 5-minute delay was used for thermal equilibration for each 2.5 K step. A complete listing of integrals is provided on Table S1. All ^{1}H-NMR spectra were obtained using a Bruker DRX 300 (^{1}H freq. = 300 MHz) with solvent d_6-DMSO.

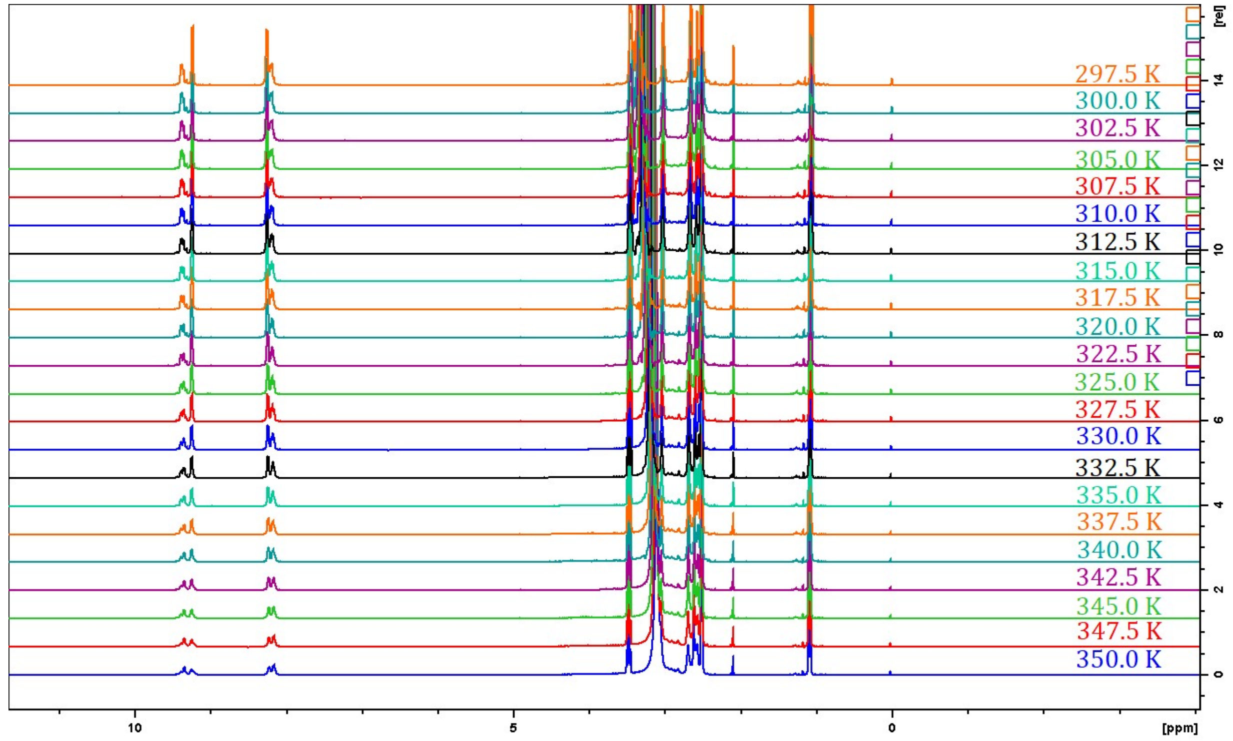

Figure S14. Full range spectra obtained over the first cooling (350.0 K, bottom to 297.5 K top), with each spectra obtained at the listed temperature. A 5-minute delay was used for thermal equilibration for each 2.5 K step. A complete listing of integrals is provided on Table S1. All ^{1}H-NMR spectra were obtained using a Bruker DRX 300 (^{1}H freq. = 300 MHz) with solvent d_6-DMSO.

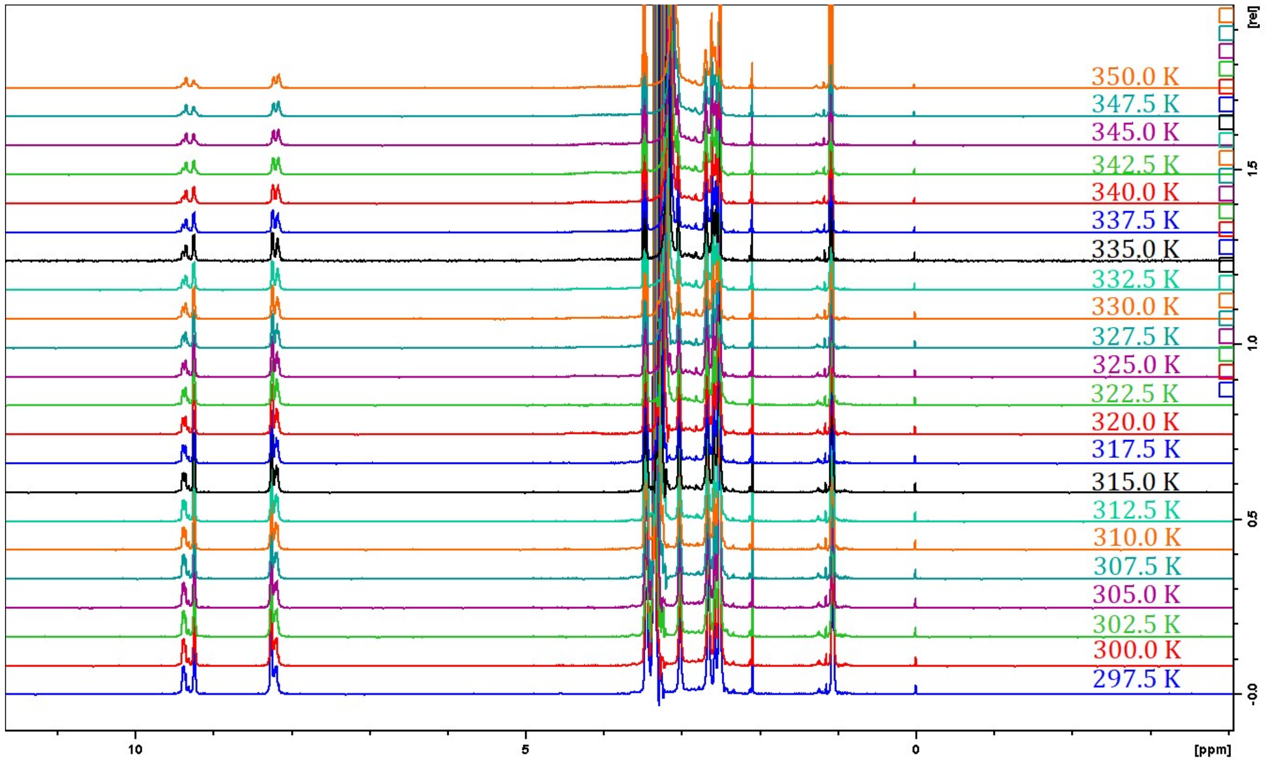

Figure S15. Full range spectra obtained over the second heating (297.5 K, bottom to 350.0 K top), with each spectra obtained at the listed temperature. A 5-minute delay was used for thermal equilibration for each 2.5 K step. A complete listing of integrals is provided on Table S1. All ^{1}H-NMR spectra were obtained using a Bruker DRX 300 (^{1}H freq. = 300 MHz) with solvent d_6-DMSO.

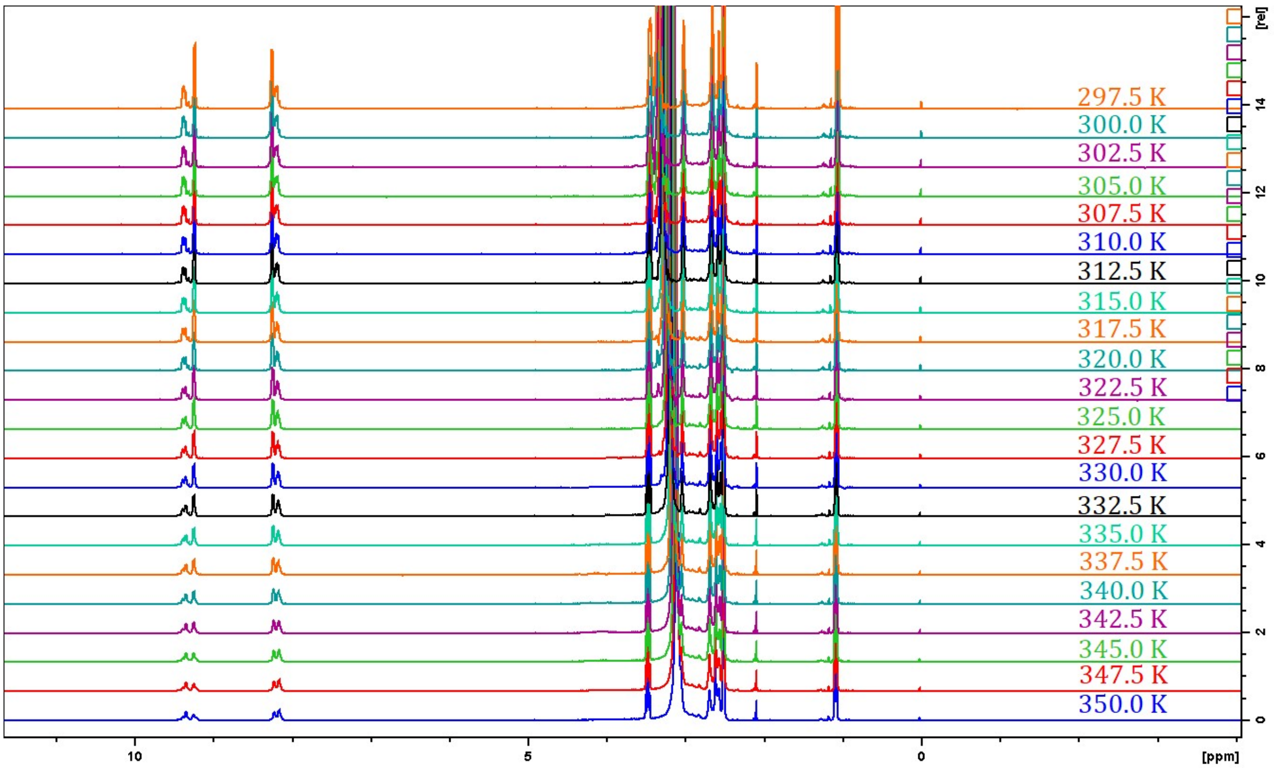

Figure S16. Full range spectra obtained over the first cooling (350.0 K, bottom to 297.5 K top), with each spectrum obtained at the listed temperature. A 5-minute delay was used for thermal equilibration for each 2.5 K step. A complete listing of integrals is provided on Table S1. All ^{1}H-NMR spectra were obtained using a Bruker DRX 300 (^{1}H freq. = 300 MHz) with solvent d_6-DMSO.

Table S1. Tabulated VT–NMR data and derived quantities in following the thermochemical analysis protocol.

Time (min)	Temp (K)	Absolute Integrals			Relative Integrals			Equilibrium Constants			*ΔG* (kcal mol^{-1})		
		**$M_3^{lin}L_3$**	**$M_4^{lin}L_4$**	**$M_2^{lin}L_3$**	**$M_3^{lin}L_3$**	**$M_4^{lin}L_4$**	**$M_2^{lin}L_3$**	**$M_3^{lin}L_3$**	**$M_4^{lin}L_4$**	**$M_2^{lin}L_3$**	**$M_3^{lin}L_3$**	**$M_4^{lin}L_4$**	**$M_2^{lin}L_3$**
0	297.5	6.96E+06	2.52E+06	2.10E+06	0.601	0.218	0.181	3.152	0.152	0.110	0.012	1.804	1.993
6	300.0	6.84E+06	2.46E+06	2.07E+06	0.602	0.216	0.182	3.178	0.149	0.111	0.001	1.822	2.000
12	302.5	6.60E+06	2.38E+06	2.03E+06	0.599	0.216	0.185	3.110	0.149	0.114	0.008	1.833	1.997
18	305.0	6.35E+06	2.32E+06	1.97E+06	0.597	0.218	0.185	3.027	0.152	0.114	0.019	1.829	2.005
24	307.5	6.06E+06	2.25E+06	1.93E+06	0.592	0.219	0.188	2.909	0.154	0.117	0.038	1.833	1.998
30	310.0	5.80E+06	2.16E+06	1.87E+06	0.590	0.220	0.190	2.856	0.154	0.119	0.044	1.840	1.999
36	312.5	5.46E+06	2.13E+06	1.82E+06	0.580	0.226	0.194	2.613	0.163	0.123	0.094	1.817	1.990
42	315.0	5.18E+06	2.02E+06	1.79E+06	0.576	0.224	0.199	2.527	0.160	0.129	0.110	1.836	1.969
48	317.5	4.90E+06	1.93E+06	1.81E+06	0.567	0.224	0.209	2.330	0.160	0.141	0.157	1.847	1.926
54	320.0	4.45E+06	1.79E+06	1.75E+06	0.557	0.224	0.219	2.142	0.160	0.153	0.206	1.856	1.883
60	322.5	3.97E+06	1.67E+06	1.69E+06	0.542	0.228	0.230	1.886	0.165	0.168	0.284	1.846	1.832
66	325.0	3.60E+06	1.58E+06	1.63E+06	0.528	0.232	0.239	1.679	0.171	0.181	0.356	1.829	1.793
72	327.5	3.28E+06	1.50E+06	1.55E+06	0.518	0.237	0.245	1.544	0.178	0.189	0.407	1.812	1.773
78	330.0	2.99E+06	1.40E+06	1.56E+06	0.503	0.235	0.262	1.361	0.175	0.218	0.488	1.833	1.688
84	332.5	2.68E+06	1.32E+06	1.52E+06	0.485	0.239	0.276	1.183	0.181	0.242	0.579	1.817	1.628
90	335.0	2.39E+06	1.25E+06	1.47E+06	0.468	0.244	0.288	1.037	0.189	0.265	0.666	1.799	1.573
96	337.5	2.13E+06	1.15E+06	1.54E+06	0.442	0.238	0.319	0.850	0.180	0.338	0.799	1.840	1.417
102	340.0	1.88E+06	1.10E+06	1.50E+06	0.419	0.246	0.335	0.711	0.191	0.381	0.920	1.808	1.341
108	342.5	1.63E+06	1.03E+06	1.42E+06	0.399	0.252	0.349	0.613	0.200	0.423	1.023	1.784	1.276
114	345.0	1.46E+06	8.92E+05	1.49E+06	0.380	0.233	0.388	0.530	0.172	0.563	1.125	1.897	1.084
120	347.5	1.21E+06	8.81E+05	1.41E+06	0.345	0.251	0.404	0.409	0.200	0.634	1.306	1.802	1.005
126	350.0	1.03E+06	8.03E+05	1.41E+06	0.318	0.247	0.435	0.333	0.193	0.804	1.454	1.832	0.842
132	347.5	1.20E+06	8.77E+05	1.39E+06	0.345	0.253	0.402	0.410	0.202	0.626	1.305	1.794	1.014
138	345.0	1.38E+06	9.18E+05	1.38E+06	0.376	0.249	0.375	0.515	0.196	0.513	1.145	1.807	1.147
144	342.5	1.61E+06	9.85E+05	1.42E+06	0.402	0.245	0.353	0.625	0.190	0.435	1.010	1.819	1.257
150	340.0	1.81E+06	1.07E+06	1.50E+06	0.413	0.245	0.342	0.681	0.190	0.400	0.949	1.813	1.308
156	337.5	2.04E+06	1.14E+06	1.46E+06	0.441	0.245	0.314	0.840	0.190	0.325	0.807	1.803	1.444
162	335.0	2.32E+06	1.21E+06	1.46E+06	0.465	0.242	0.293	1.009	0.185	0.277	0.684	1.811	1.544
168	332.5	2.57E+06	1.30E+06	1.46E+06	0.482	0.244	0.274	1.158	0.188	0.238	0.593	1.793	1.637
174	330.0	2.83E+06	1.38E+06	1.44E+06	0.501	0.244	0.255	1.345	0.188	0.205	0.496	1.784	1.728
180	327.5	3.15E+06	1.43E+06	1.50E+06	0.518	0.236	0.246	1.543	0.176	0.192	0.408	1.820	1.765
186	325.0	3.43E+06	1.53E+06	1.54E+06	0.528	0.236	0.237	1.668	0.176	0.177	0.360	1.812	1.806
192	322.5	3.75E+06	1.62E+06	1.55E+06	0.542	0.234	0.224	1.885	0.173	0.160	0.284	1.814	1.864
198	320.0	4.12E+06	1.69E+06	1.67E+06	0.551	0.226	0.223	2.028	0.162	0.159	0.241	1.845	1.860
204	317.5	4.39E+06	1.80E+06	1.69E+06	0.557	0.229	0.215	2.128	0.166	0.148	0.214	1.821	1.897
210	315.0	4.70E+06	1.89E+06	1.67E+06	0.569	0.228	0.202	2.379	0.166	0.133	0.148	1.814	1.954
216	312.5	5.05E+06	1.98E+06	1.80E+06	0.572	0.224	0.204	2.427	0.160	0.135	0.140	1.827	1.933
222	310.0	5.36E+06	2.06E+06	1.83E+06	0.580	0.222	0.198	2.600	0.157	0.128	0.102	1.829	1.954
228	307.5	5.66E+06	2.13E+06	1.95E+06	0.581	0.219	0.200	2.634	0.153	0.130	0.098	1.835	1.936
234	305.0	5.93E+06	2.24E+06	1.97E+06	0.585	0.221	0.194	2.725	0.156	0.124	0.083	1.816	1.956
240	302.5	6.20E+06	2.35E+06	2.00E+06	0.588	0.223	0.189	2.804	0.158	0.119	0.071	1.799	1.971
246	300.0	6.45E+06	2.40E+06	2.12E+06	0.588	0.218	0.193	2.810	0.153	0.123	0.074	1.810	1.940
252	297.5	6.53E+06	2.48E+06	2.03E+06	0.591	0.225	0.184	2.885	0.161	0.113	0.064	1.769	1.980
258	300.0	6.46E+06	2.43E+06	2.06E+06	0.590	0.222	0.188	2.849	0.158	0.117	0.066	1.791	1.969

264	302.5	6.24E+06	2.40E+06	1.95E+06	0.589	0.227	0.184	2.825	0.164	0.113	0.066	1.778	1.999
270	305.0	6.02E+06	2.31E+06	1.96E+06	0.585	0.224	0.190	2.733	0.160	0.120	0.081	1.799	1.976
276	307.5	5.78E+06	2.21E+06	1.98E+06	0.580	0.222	0.199	2.605	0.157	0.129	0.105	1.822	1.942
282	310.0	5.53E+06	2.11E+06	1.92E+06	0.578	0.221	0.201	2.565	0.156	0.131	0.110	1.834	1.940
288	312.5	5.18E+06	2.07E+06	1.78E+06	0.574	0.229	0.197	2.470	0.167	0.127	0.129	1.802	1.970
294	315.0	4.88E+06	1.95E+06	1.76E+06	0.568	0.227	0.205	2.346	0.164	0.136	0.157	1.821	1.938
300	317.5	4.58E+06	1.85E+06	1.77E+06	0.558	0.226	0.216	2.156	0.162	0.149	0.206	1.837	1.888
306	320.0	4.20E+06	1.77E+06	1.64E+06	0.552	0.233	0.215	2.039	0.172	0.148	0.237	1.807	1.902
312	322.5	3.86E+06	1.70E+06	1.64E+06	0.536	0.236	0.228	1.789	0.176	0.166	0.318	1.803	1.841
318	325.0	3.58E+06	1.56E+06	1.65E+06	0.527	0.229	0.243	1.665	0.167	0.187	0.361	1.844	1.772
324	327.5	3.24E+06	1.52E+06	1.57E+06	0.512	0.240	0.248	1.467	0.182	0.195	0.441	1.799	1.753
330	330.0	2.95E+06	1.40E+06	1.57E+06	0.499	0.236	0.265	1.322	0.177	0.222	0.507	1.825	1.677
336	332.5	2.61E+06	1.35E+06	1.48E+06	0.480	0.248	0.272	1.139	0.195	0.234	0.604	1.770	1.648
342	335.0	2.34E+06	1.25E+06	1.45E+06	0.463	0.249	0.288	1.000	0.196	0.265	0.690	1.775	1.573
348	337.5	2.10E+06	1.15E+06	1.50E+06	0.442	0.243	0.315	0.849	0.186	0.328	0.800	1.817	1.438
354	340.0	1.82E+06	1.10E+06	1.47E+06	0.415	0.251	0.334	0.691	0.199	0.378	0.940	1.781	1.347
360	342.5	1.59E+06	1.01E+06	1.49E+06	0.388	0.247	0.365	0.565	0.192	0.476	1.078	1.811	1.196
366	345.0	1.41E+06	9.34E+05	1.45E+06	0.372	0.246	0.382	0.499	0.192	0.539	1.166	1.821	1.113
372	347.5	1.23E+06	8.60E+05	1.46E+06	0.346	0.242	0.412	0.414	0.185	0.674	1.299	1.855	0.962
378	350.0	1.05E+06	7.59E+05	1.45E+06	0.322	0.233	0.445	0.345	0.172	0.867	1.430	1.914	0.789
384	347.5	1.19E+06	8.52E+05	1.41E+06	0.345	0.247	0.408	0.408	0.193	0.658	1.309	1.826	0.979
390	345.0	1.35E+06	9.16E+05	1.40E+06	0.368	0.250	0.382	0.487	0.198	0.538	1.183	1.801	1.115
396	342.5	1.58E+06	9.95E+05	1.39E+06	0.399	0.251	0.350	0.612	0.199	0.425	1.024	1.787	1.272
402	340.0	1.77E+06	1.08E+06	1.38E+06	0.417	0.256	0.327	0.704	0.207	0.357	0.927	1.754	1.385
408	337.5	2.06E+06	1.12E+06	1.47E+06	0.443	0.241	0.316	0.853	0.183	0.330	0.797	1.827	1.432
414	335.0	2.30E+06	1.22E+06	1.48E+06	0.460	0.244	0.296	0.974	0.189	0.282	0.708	1.800	1.532
420	332.5	2.56E+06	1.29E+06	1.43E+06	0.485	0.245	0.270	1.182	0.189	0.232	0.579	1.789	1.654
426	330.0	2.83E+06	1.34E+06	1.49E+06	0.500	0.236	0.263	1.337	0.177	0.219	0.499	1.825	1.684
432	327.5	3.10E+06	1.45E+06	1.50E+06	0.513	0.239	0.248	1.485	0.181	0.194	0.433	1.803	1.757
438	325.0	3.41E+06	1.52E+06	1.50E+06	0.531	0.236	0.233	1.711	0.177	0.172	0.343	1.808	1.826
444	322.5	3.70E+06	1.63E+06	1.56E+06	0.537	0.236	0.226	1.808	0.177	0.163	0.311	1.800	1.851
450	320.0	4.01E+06	1.71E+06	1.59E+06	0.549	0.234	0.218	1.989	0.173	0.152	0.253	1.805	1.888
456	317.5	4.34E+06	1.79E+06	1.66E+06	0.558	0.230	0.213	2.148	0.168	0.145	0.208	1.817	1.907
462	315.0	4.64E+06	1.91E+06	1.68E+06	0.564	0.232	0.204	2.265	0.171	0.135	0.179	1.796	1.943
468	312.5	4.98E+06	2.00E+06	1.74E+06	0.571	0.230	0.199	2.412	0.167	0.129	0.144	1.799	1.959
474	310.0	5.28E+06	2.07E+06	1.84E+06	0.574	0.226	0.200	2.474	0.162	0.131	0.132	1.810	1.943
480	307.5	5.60E+06	2.15E+06	1.84E+06	0.584	0.224	0.192	2.708	0.160	0.121	0.082	1.811	1.978
486	305.0	5.88E+06	2.25E+06	1.98E+06	0.582	0.222	0.196	2.654	0.158	0.125	0.099	1.809	1.948
492	302.5	6.15E+06	2.35E+06	1.93E+06	0.590	0.225	0.185	2.853	0.162	0.113	0.060	1.784	1.997
498	300.0	6.43E+06	2.41E+06	2.08E+06	0.589	0.221	0.191	2.821	0.155	0.120	0.072	1.799	1.954
504	297.5	6.48E+06	2.47E+06	2.08E+06	0.588	0.224	0.188	2.800	0.160	0.117	0.082	1.774	1.956
510	297.5	6.55E+06	2.49E+06	2.05E+06	0.591	0.224	0.185	2.873	0.160	0.114	0.066	1.773	1.973

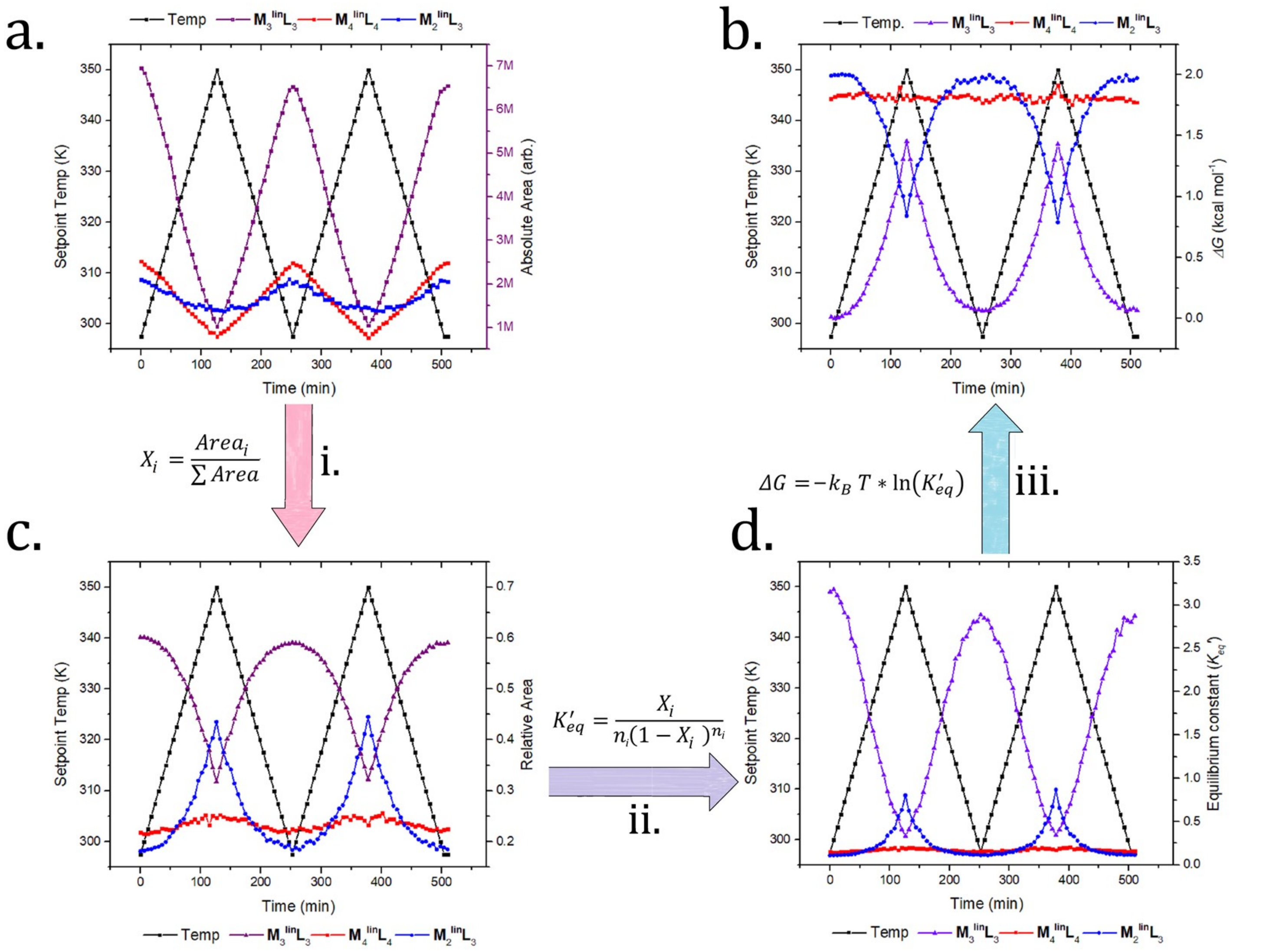

Figure S17. Plots of the VT-NMR data acquired as a function of analysis time (Table S1), shown following the thermochemical analysis (Scheme S2), listed in order of execution. (a) Absolute peak areas obtained by integration of ^{1}H-NMR spectra Figure (S12). (c) Relative peak areas obtained by (i). (d) Equilibrium constants, K_{eq}', obtained by (ii). (b) Gibbs free energies (ΔG) obtained from the Boltzmann equation (iii).

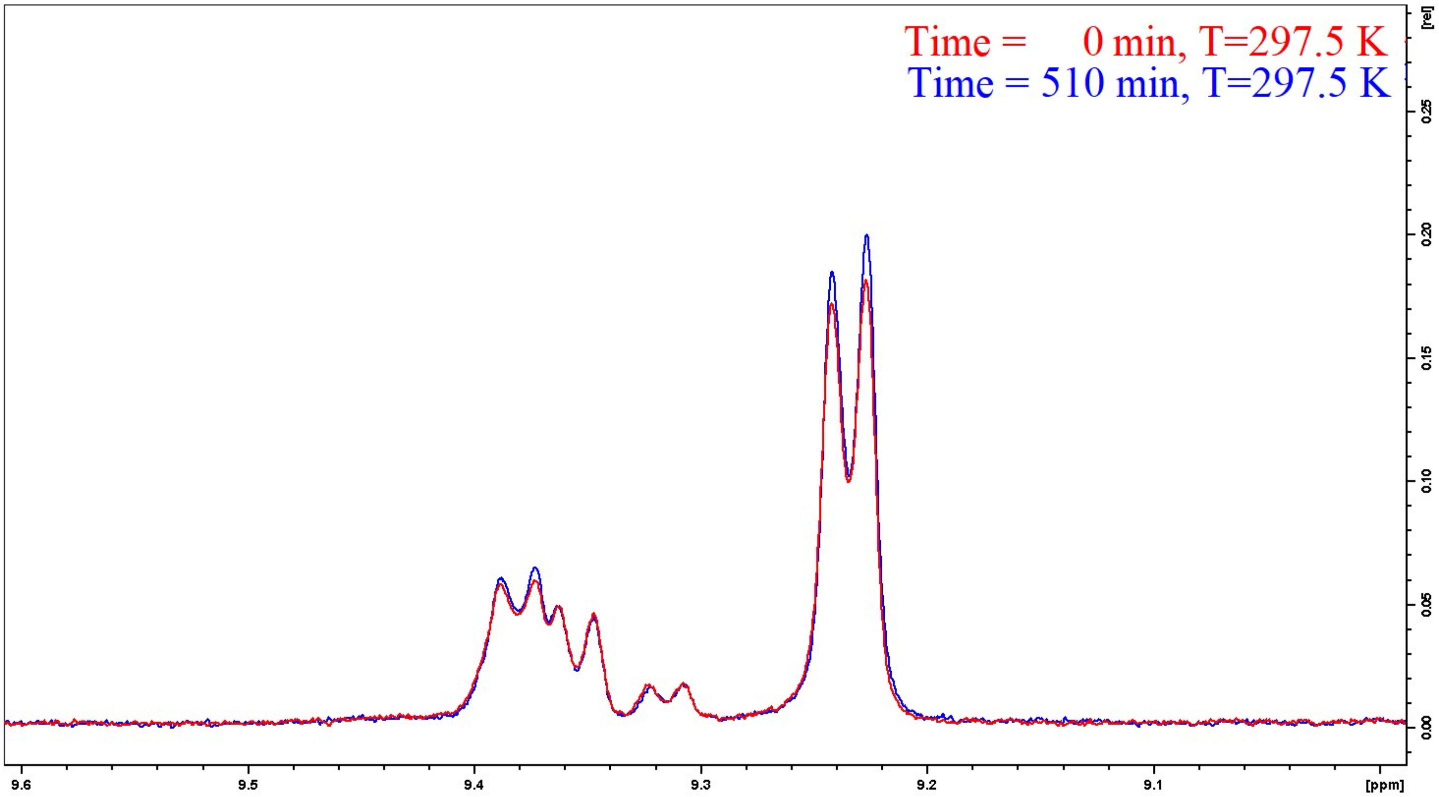

Figure S18. Overlay of the first and last 1H-NMR spectra obtained from VT-NMR showing differences in the observed peak areas for $\mathbf{M}_3{}^{\mathbf{lin}}\mathbf{L}_3$ (6.9%) and $\mathbf{M}_4{}^{\mathbf{lin}}\mathbf{L}_4$ (2.1%)

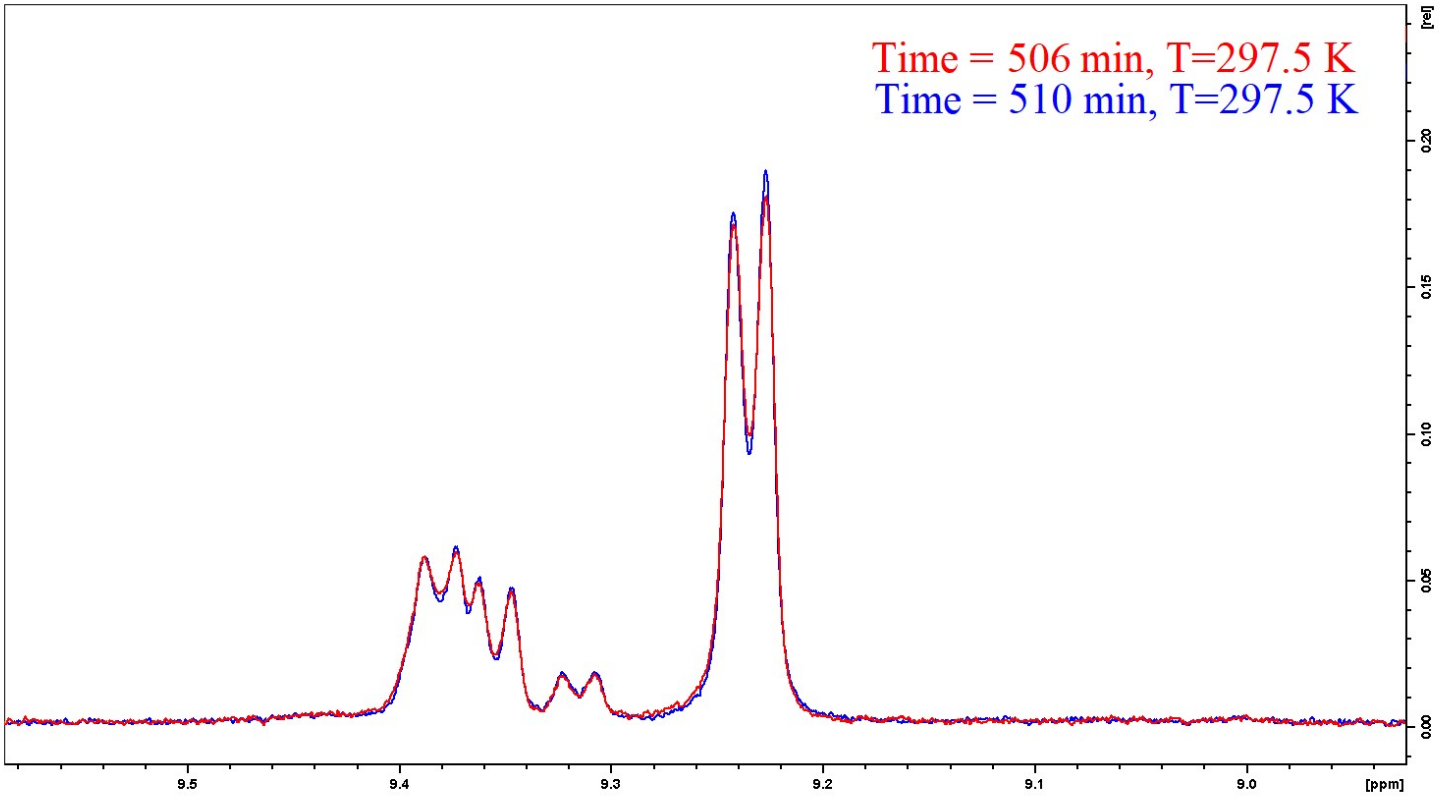

Figure S19. Overlay of the penultimate and last 1H-NMR spectra obtained from VT-NMR showing slight differences in the observed peak areas due residual temperature differences for $\mathbf{M}_3{}^{\mathbf{lin}}\mathbf{L}_3$ (1.1%) and $\mathbf{M}_4{}^{\mathbf{lin}}\mathbf{L}_4$ (0.7%).

11. Graphical explanation of *ΔH* and -*TΔS* reference states

When comparing thermodynamic data arising from different measurements (i.e., Molecular Dynamics, VT-NMR and UV–Vis), it is critical to determine the ***relative*** difference between the reference states of each measurements in ***absolute*** terms. Since the ***absolute*** enthalpy (H) and ***absolute*** entropy (-TS) are orthogonal, non-interacting dimensions (i.e., a change in ***absolute*** enthalpy cannot influence ***absolute*** entropy), this comparison can be made geometrically.

- Figure S20a depicts how one can translate a single thermodynamic measurement from a ***reference*** ($\mathbf{Ref_1}$) to a ***state*** ($\mathbf{S_1}$) in a two-dimensional Enthalpy–Entropy plot, with the ***absolute*** enthalpy (H) on the *x*-axis and ***absolute*** entropy (-TS) on the *y*-axis.

- Figure S20b depicts an Enthalpy–Entropy plot of the ***absolute*** enthalpy and ***absolute*** entropy for three measured states (i.e., measurements of three different samples, $\mathbf{S_1}$, $\mathbf{S_2}$, $\mathbf{S_3}$, in ***absolute*** H and -TS terms). Note, in this situation the respective positions of the measured states ($\mathbf{S_1}$, $\mathbf{S_2}$, $\mathbf{S_3}$) relative to each other does not change even if the position of the reference ($\mathbf{Ref_1}$) does.

- Figure S20c shows the scenario the point of reference has been moved from $\mathbf{Ref_1}$ (0,0) to $\mathbf{Ref_2}$ (2,-1), where the three measured states ($\mathbf{S_1}$, $\mathbf{S_2}$, $\mathbf{S_3}$) were measured with a new reference (e.g., arising from instrumental drift). Effectively, we can ***translate*** (but not rotate) these measured states arbitrarily, as the respective positions of the measured states ($\mathbf{S_1}$, $\mathbf{S_2}$, $\mathbf{S_3}$) relative to each other does not change, even if the position of the reference ($\mathbf{Ref_1}$) does.

- Figure S20d shows how this geometric representation can be applied to our experimental system. We can ***translate*** the MD-measured thermodynamic data to overlap with the VT-NMR data using the respective centroid of the three measured states ($\mathbf{S_1}$, $\mathbf{S_2}$, $\mathbf{S_3}$) from each experiment.

- This technique requires at least two measured states (e.g., a control and observation measurement), but graphically demonstrates the differences observed in our data in ***absolute*** terms. From this comparison we see that ***absolute*** differences between VT-NMR and MD values of H and -TS for the three measured states $\mathbf{M}_3^{\mathbf{lin}}\mathbf{L}_3$, $\mathbf{M}_4^{\mathbf{lin}}\mathbf{L}_4$, $\mathbf{M}_2^{\mathbf{lin}}\mathbf{L}_3$ are smaller than the ***absolute*** H and -TS differences between the individual assemblies, indicating that the approaches are in good agreement. Specifically, the largest difference between VT-NMR and MD approaches for a single measured state ($\mathbf{D_1}$, for $\mathbf{M}_3^{\mathbf{lin}}\mathbf{L}_3$) is smaller than the smallest difference between any two measured states taken with a single measurement approach ($\mathbf{D_2}$, $\mathbf{M}_3^{\mathbf{lin}}\mathbf{L}_3$ and $\mathbf{M}_4^{\mathbf{lin}}\mathbf{L}_4$ measured by MD).

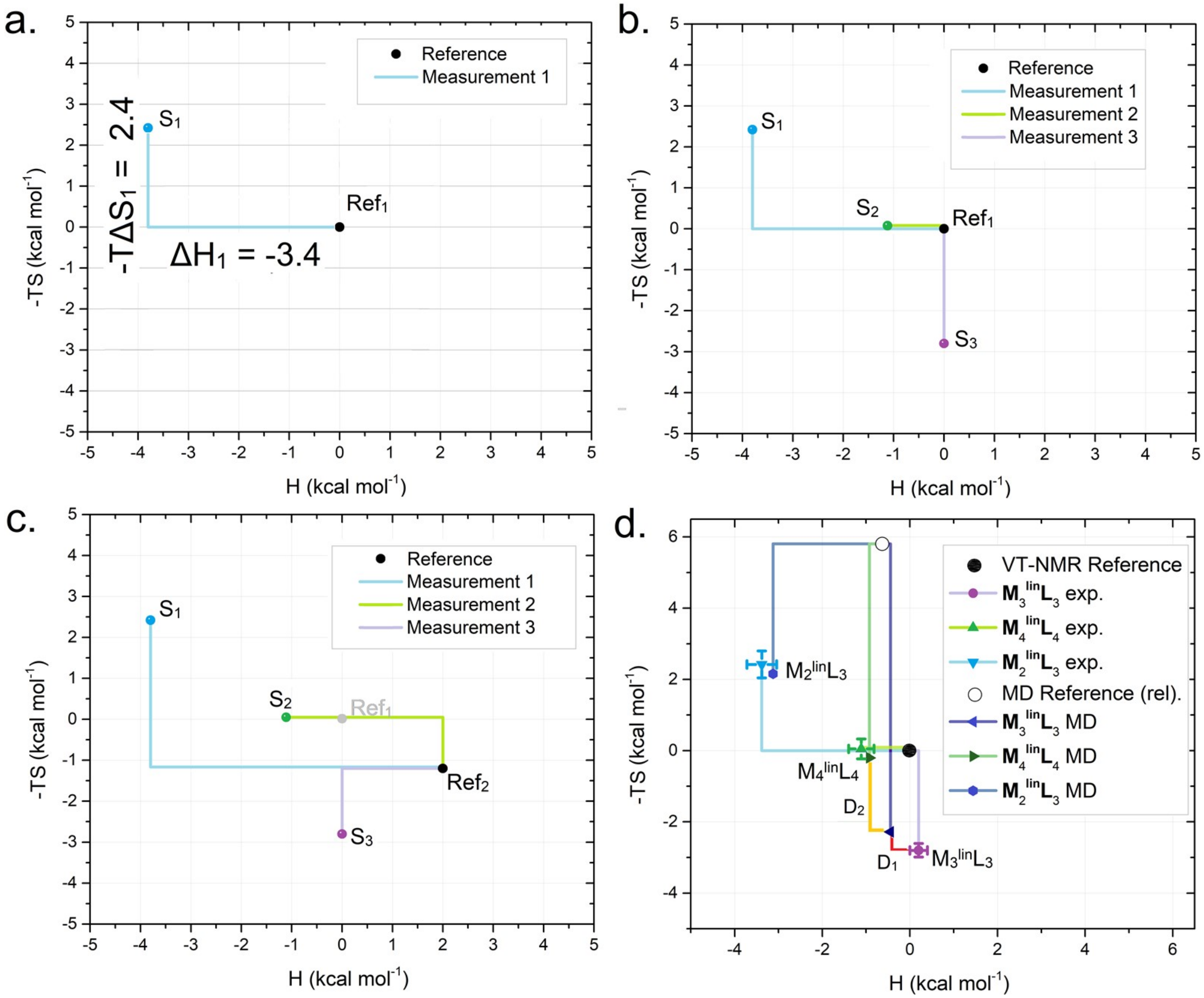

Figure S20. A geometric representation of measured thermodynamic data in H-TS space. (a) An example of a single measured state (S_1) from some reference (**Ref$_1$**). (b) An example of three measured states (**S$_1$**, **S$_2$**, **S$_3$**) from a single reference point (**Ref$_1$**). (c) An example of the same states (**S$_1$**, **S$_2$**, **S$_3$**), but measured from a reference (**Ref$_2$**, in black) that is offset from the previous reference (**Ref$_1$**, in grey). (d) A comparison of VT-NMR and MD data, referenced to the VT-NMR, showing the largest difference between VT-NMR and MD approaches (**D$_1$**) and the smallest difference between measured states (**D$_2$**).

12. References

1. M. Fujita, J. Yazaki, and K. Ogura, *J. Am. Chem. Soc.* 1990, 112, 14, 5645–5647, doi: 10.1021/ja00170a042.
2. D. A. Poole, III., E.O. Bobylev, S. Mathew, and J. N. H. Reek, J. N. H. *Chem. Sci.* 2020, **11**, 12350–12357, doi: 10.1039/D0SC03992F.
3. C. Bannwarth, S. Ehlert, and S. Grimme, *J. Chem. Theory Comput.* 2019, **15** (3), 1652–1671, DOI: 10.1021/acs.jctc.8b01176.
4. M. J. Frisch, G. W. Trucks, H. B. Schlegel, G. E. Scuseria, M. A. Robb, J. R. Cheeseman, G. Scalmani, V. Barone, G. A. Petersson, H. Nakatsuji, X. Li, M. Caricato, A. V. Marenich, J. Bloino, B. G. Janesko, R. Gomperts, B. Mennucci, H. P. Hratchian, J. V. Ortiz, A. F. Izmaylov, J. L. Sonnenberg, D. Williams-Young, F. Ding, F. Lipparini, F. Egidi, J. Goings, B. Peng, A. Petrone, T. Henderson, D. Ranasinghe, V. G. Zakrzewski, J. Gao, N Rega, G. Zheng, W. Liang, M. Hada, M. Ehara, K. Toyota, R. Fukuda, J. Hasegawa, M. Ishida, T. Nakajima, Y. Honda, O. Kitao, H. Nakai, T. Vreven, K. Throssell, J. A. Montgomery, Jr., J. E. Peralta, F. Ogliaro, M. J. Bearpark, J. J. Heyd, E. N. Brothers, K. N. Kudin, V. N. Staroverov, T. A. Keith, R. Kobayashi, J. Normand, K. Raghavachari, A. P. Rendell, J. C. Burant, S. S. Iyengar, J. Tomasi, M. Cossi, J. M. Millam, M. Klene, C. Adamo, R. Cammi, J. W. Ochterski, R. L. Martin, K. Morokuma, O. Farkas, J. B. Foresman, and D. J. Fox, Gaussian 16 Revision C.01, Gaussian Inc., Wallingford CT, 2016.
5. R. M. Betz and R. C. Walker, *J. Comput. Chem.* 2014, **36**(2), 79–87, DOI:10.1002/jcc.23775.
6. D. A. Case, D. S. Cerutti, T. E. Cheatham, III, T. A. Darden, R. E. Duke, T. J. Giese, H. Gohlke, A. W. Goetz, S. Izadi, P. Janowski, J. Kaus, A. Kovalenko, T. S. Lee, S. LeGrand, C. Li, T. L. Lin, R. Luo, B. Madej, D. Mermelstein, K. M. Merz, G. Monard, H. Nguyen, I. Nguyen, A. O. Omelyan, D. R. Roe, A. Roitberg, C. Sagui, C. L. Simmerling, W. M. Botello-Smith, J. Swails, J. W. Walker, R. M. Wolf, X. Wu, L. Xiao, and P. A. Kollman, AMBER 2016, University of California, San Francisco, 2016.
7. R. Salomon-Ferrer, A. W. Goetz, D. Poole, S. Le Grand, and R. C. Walker, J. *Chem. Theory Comput.* 2013, **9**, 3878–3888.
8. D. R. Roe and T. E. Cheatham, T. E. *J. Chem. Theory Comput.* 2013, **9**(7), 3084–3095. doi:10.1021/ct400341p.
9. R. M. Levy, M. Karplus, J. Kushick, and D. Perahia, *Macromolecules* 1984, **17**, 7, 1370–1374, doi: 10.1021/ma00137a013.
10. C. G. Maier and K. K. Kelly, *J. Am. Chem. Soc.*, 1932, **54**, 8, 3243–3246, doi: 10.1021/ja01347a029.
11. T. Weilandt, N. L. Löw, G. Schnakenburg, J. Daniels, M. Nieger, C. A. Schalley and A. Lützen, *Chem. Eur. J.,* 2012, **18**, 52, 16665–16676, doi:10.1002/chem.201202771.

.
